# 6-Shogaol Derived from Ginger Inhibits Intestinal Crypt Stem Cell Differentiation and Contributes to Irritable Bowel Syndrome Risk

**DOI:** 10.34133/research.0524

**Published:** 2024-11-07

**Authors:** Bing Zhao, Juan Ye, Wenjing Zhao, Xinyu Liu, Hongli Lan, Jinbing Sun, Jiao Chen, Xueting Cai, Qingyun Wei, Qian Zhou, Zhengwei Zhang, Yuze Wu, Yang Yang, Peng Cao

**Affiliations:** ^1^ State Key Laboratory on Technologies for Chinese Medicine Pharmaceutical Process Control and Intelligent Manufacture, Nanjing University of Chinese Medicine, Nanjing, China.; ^2^ Jiangsu Provincial Medical Innovation Center, Affiliated Hospital of Integrated Traditional Chinese and Western Medicine, Nanjing University of Chinese Medicine, Nanjing, China.; ^3^ Department of General Surgery, Changshu No. 1 People’s Hospital, Affiliated Changshu Hospital of Soochow University, Changshu, China.

## Abstract

Dietary factors play a crucial role in irritable bowel syndrome (IBS) pathogenesis. Therefore, the dietary contraindications for patients with IBS require further supplementation. Recent investigations have revealed that ginger consumption may pose a risk of aggravating the symptoms and incidence of IBS; however, the specific mechanism remains unknown. In this study, we developed experimental IBS and intestinal organoid differentiation screening models to elucidate the mechanisms underlying the ginger-mediated exacerbation of IBS symptoms. Subsequently, we used a knockout approach combined with click chemistry as well as virus infection to identify the toxic components of ginger and the target mechanism. Our results showed that a daily intake of 90 to 300 mg/kg ginger (equivalent to a human daily dose of 0.6 to 2 g per person) may pose a risk of exacerbating IBS symptoms. Furthermore, a component derived from 6-gingerol (ginger’s main ingredient) through in vivo gastric acid and heat processing inhibited the formation of the eIF3 transcription initiation complex by covalently binding to the Cys^58^ site of eIF3A, a key factor regulating intestinal crypt stem cell differentiation, further reducing the goblet cell number and related mucus layer thickness and increasing lipopolysaccharide infiltration and low-grade inflammation in the ileum crypts, thereby exacerbating the symptoms of IBS in mice. Our study suggests that dietary ginger aggravates IBS and provides safety evaluation methods for the proper use of foods in specific populations.

## Introduction

Irritable bowel syndrome (IBS) is a common chronic bowel disorder estimated to affect approximately 1 in 10 people globally [1]. Dietary factors play an important role in triggering or exacerbating IBS, with approximately ^2^/_3_ of patients with IBS reporting worsening symptoms after meals [2], and approximately 90% of patients with IBS rely on exclusion diets to prevent or alleviate gastrointestinal symptoms [3]. Thus, the aggravated role of dietary factors in IBS has been gaining attention; however, the dietary contraindications table for patients with IBS requires continuous revision. In addition to understanding gluten (including wheat)- and fermentable oligo-di-monosaccharide and polyol (FODMAP)-containing food sensitivity in IBS symptoms [4], the material basis and mechanism of IBS symptoms caused by daily rest foods is important for improving exclusion diet formulation for patients with IBS.

Ginger (*Zingiber officinale* Roscoe; Zingiberaceae), the rhizome, is one of the most commonly consumed spices and flavoring agents worldwide and is considered a low-FODMAP substitute for patients with IBS [3,5]. However, the application of ginger in patients with IBS remains controversial. From the perspective of countries with high consumption of ginger [6], there is a relatively high incidence rate of IBS under Rome IV and II Rome criteria (particularly in the main Asian and African consumer countries of ginger, such as Bangladesh, Indonesia, and Nigeria), but its correlation is rarely studied [7,8]. A comprehensive survey examining the relationship between food consumption and IBS discovered that women who consume spicy foods such as pepper, curry, saffron, ginger, cinnamon, and turkey more than 10 times a week are about twice as likely to develop IBS [9]. Moreover, IBS-related inflammatory activation has been reported to be high in a ginger-consumption group and among those consuming wheat and certain high-FODMAP foods [10]. Ginger was used as a remedy for IBS in a large survey of patients with diarrhea-predominant IBS [11,12]. Nevertheless, the use of ginger to treat IBS remains debated. In a small-scale, double-blind, randomized, controlled pilot study involving 45 subjects with ginger application in IBS patients, ginger did not perform better than placebo, with lower responses and higher IBS severity scores observed in the ginger treatment than placebo groups [13]. In the European Union, ginger has been approved for treating gastrointestinal diseases [12]; however, the adverse effects analysis of randomized controlled clinical trials on ginger showed that daily intake of ginger (750 mg to 1 g) induces intestinal adverse reactions (2% to 10%), such as diarrhea and abdominal pain in subjects [14]. Therefore, the current research on ginger and IBS has yielded conflicting results due to, among others, heterogeneity of the population, lenient inclusion criteria, lack of comprehensive evaluation, and small study size. This necessitates further research on the effects of ginger on IBS to provide an evidence-based reference for physicians to optimize its use in clinical settings.

Patients with IBS frequently struggle with establishing an effective exclusive diet for symptom alleviation [15]. This is because the medical community lacks sufficient data correlating IBS symptoms with specific foods [3,4]. Moreover, one of the reasons for the controversy surrounding the efficacy of ginger, which is widely used as a spice and herb by patients with IBS, is the lack of awareness about its components and the pathogenesis of symptom aggravation. Thus, we explored ginger-induced IBS in vivo by constructing an experimental model and elucidated the mechanisms, components, and targets of ginger-induced side effects using an intestinal organoid model. Our study provides a new reference for developing an exclusion dietary management strategy for patients with IBS; we expect our findings to strengthen research focus on the safety evaluation of the use of proper foods in specific populations.

## Results

### Dietary ginger intake aggravates IBS symptoms in an experimental IBS model

**Fig. 1.**
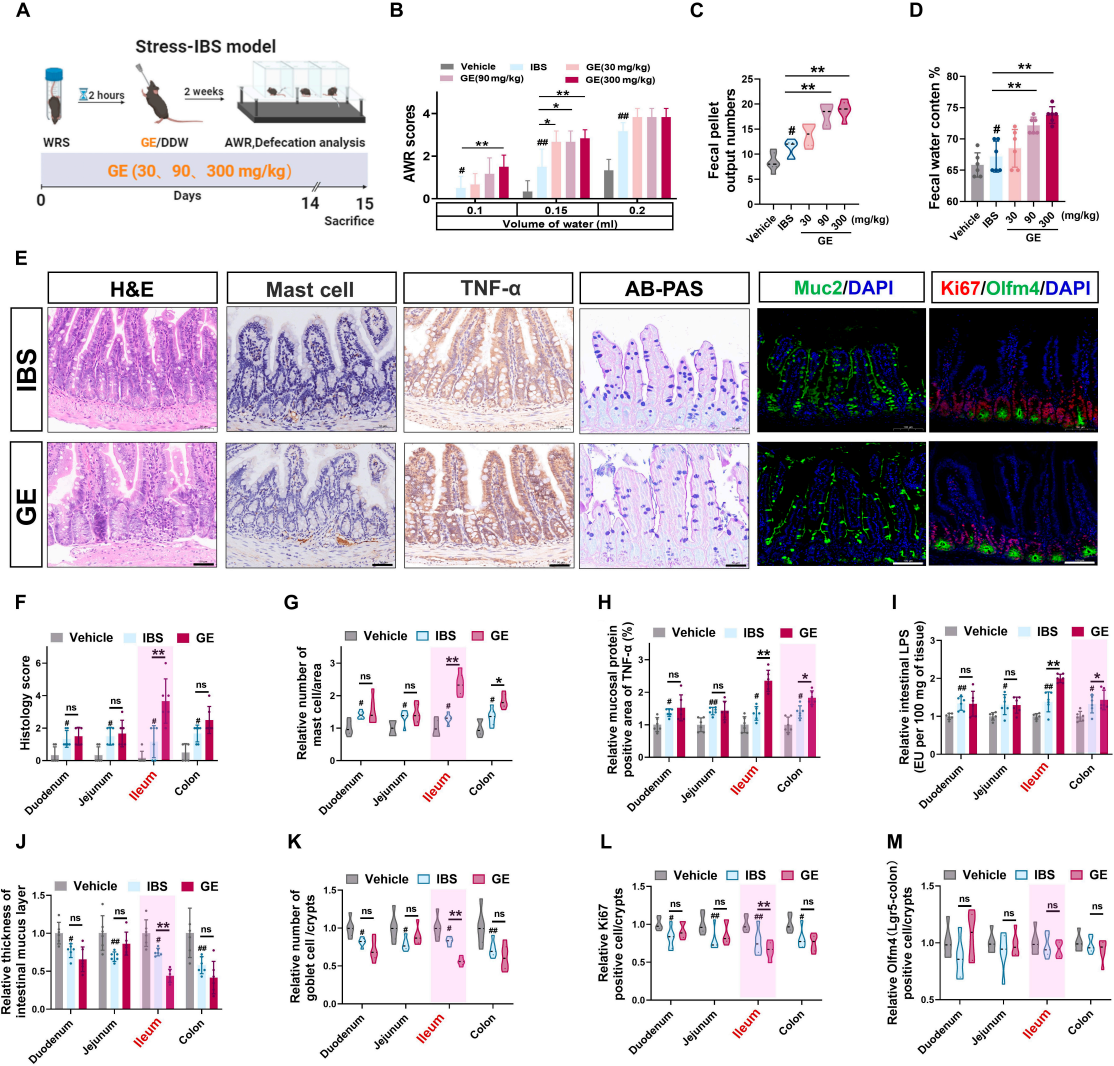
Dietary ginger aggravates IBS symptoms by inhibiting crypt stem cell differentiation and exacerbating intestinal inflammation. (A) Flow chart of the stress-induced IBS-application model to detect the IBS-aggravating effect of daily ginger consumption. Comparative analysis of IBS disease index: (B) AWR score, (C) number of fecal pellets, and (D) fecal water content of mice after gavage with different doses of ginger (according to human daily per capita intake). (E) Hematoxylin and eosin (H&E) and Alcian blue–periodic acid-Schiff (AB-PAS) staining analysis of mouse intestinal tissues (ileum); immunohistochemical analysis of mast cell and TNF-α; immunofluorescence analysis of mouse intestinal tissues for MUC2, Ki67, and Olfm4 in IBS and GE (300 mg/kg) groups. Scale bar (black), 50 μm; scale bar (white), 100 μm. Comparative analysis of different intestinal sections (duodenum, jejunum, ileum, and colon): (F) histology score; (G) mast cell infiltration; (H) TNF-α abundance; (I) intestinal LPS level; (J) thickness of intestinal mucus layer; mean number of (K) goblet cells, (L) Ki67, and (M) Olfm4 [or leucine-rich repeat containing G protein-coupled receptor 5 (Lgr5)]-positive cells per unit intestinal crypt in vehicle, IBS, and GE (300 mg/kg) groups. Data are expressed as mean ± standard deviation (*n* = 6). *P < 0.05, **P < 0.01, as indicated; ^#^ *P* < 0.05, ^##^ *P* < 0.01, compared to vehicle. DDW, double-distilled water; WRS, wrap restraint stress.

We evaluated the correlation between the global incidence rate of IBS according to different diagnostic criteria and IBS-related food intake [6–8]. We found that the amount of dietary ginger intake was positively correlated with the incidence rate of IBS under the Rome II and IV criteria, especially in some Asian and African countries (Fig. S1 and Table S1). Accordingly, we established 2 experimental IBS mouse models (stress-IBS and pro-inflammation) to verify the exacerbating effects of ginger (Fig. 1A and Fig. S2A and E). IBS is a functional gastrointestinal disorder with symptoms, including abdominal pain, associated with a change in stool form or frequency [16]. Based on the 0.2 to 2 g/60 kg per person daily ginger consumption amount from daily consumption data and dosage in ginger clinical trials [6,14], we constructed a gavage experiment in mice with a conversion dose of 30 to 300 mg/kg (original fresh ginger quantity) of ginger [ginger extract (GE) and ginger powder (GP)] to study the in vivo effect of ginger on exacerbating IBS. We found that the abdominal withdrawal reflex (AWR) score, number of defecation particles, and fecal water content of mice were increased and showed a certain dose dependence after the ingestion of >90 mg/kg of ginger (equivalent to a human daily dose of 0.6 g per person) and present corresponding dose dependence compared with those in the IBS group (Fig. 1B to D and Fig. S2B to D and F to H). Furthermore, dietary ginger intake (over 90 mg/kg equivalent to a human daily dose of 0.6 g/60 kg per person) increased the intestinal disease activity index in chemotherapy (oxaliplatin)-induced irritable bowel model (diarrhea) (Fig. S2I to K) and chronic colitis mouse model (Fig. S2L to N). These results suggest that daily intake of >90 mg/kg ginger (equivalent to a human daily dose 0.6 to 2 g per person) may pose a risk of exacerbating IBS symptoms.

### Dietary ginger exacerbates intestinal inflammatory infiltration and inhibits crypt stem cell differentiation in mice

We analyzed the pathological basis of dietary ginger-induced IBS aggravation. Based on the inflammatory activation symptoms of patients with IBS consuming ginger [10], after comparing the pathology of the intestinal segments (duodenum, jejunum, ileum, and colon), we found that ginger intake aggravated inflammatory infiltration of the small intestine (ileum) in mice (Fig. 1E and F and Figs. S3A to C and S4A), including a dose-dependent increase in the number of IBS-related mast cells in the intestinal mucosal area and level of tumor necrosis factor-α (TNF-α) in the mucosal area after inflammatory activation (Fig. 1G and H and Fig. S4B and C). Furthermore, we found that mucus thickness decreased, accompanied by reduced goblet cells and lipopolysaccharide (LPS) intestinal infiltration after ginger intake (Fig. 1I to K and Fig. S4D to F). These results suggest that ginger inhibits goblet cell regeneration, which may promote inflammatory infiltration by affecting the intestinal mucus barrier [17]. Additionally, immunofluorescence analysis revealed that ginger intake markedly reduced the number of Ki67 proliferating cells (transit-amplifying zone-Ki67^+^) in the intestinal recess but did not affect the number of olfactomedin 4 (Olfm4)-positive stem cells in the intestinal recess (Fig. 1L and M and Figs. S3C and S4G and H). These results suggest that dietary ginger inhibits ileum crypt stem cell differentiation, further reducing the goblet cell number and its secretion function, which is related to the mucus layer thickness, resulting in increased LPS infiltration and inflammation (due to mast cell and inflammatory cytokines), thereby exacerbating IBS symptoms.

### Intestinal organoid differentiation model indicates that 6-shogaol is the main IBS-aggravating component of dietary ginger

**Fig. 2.**
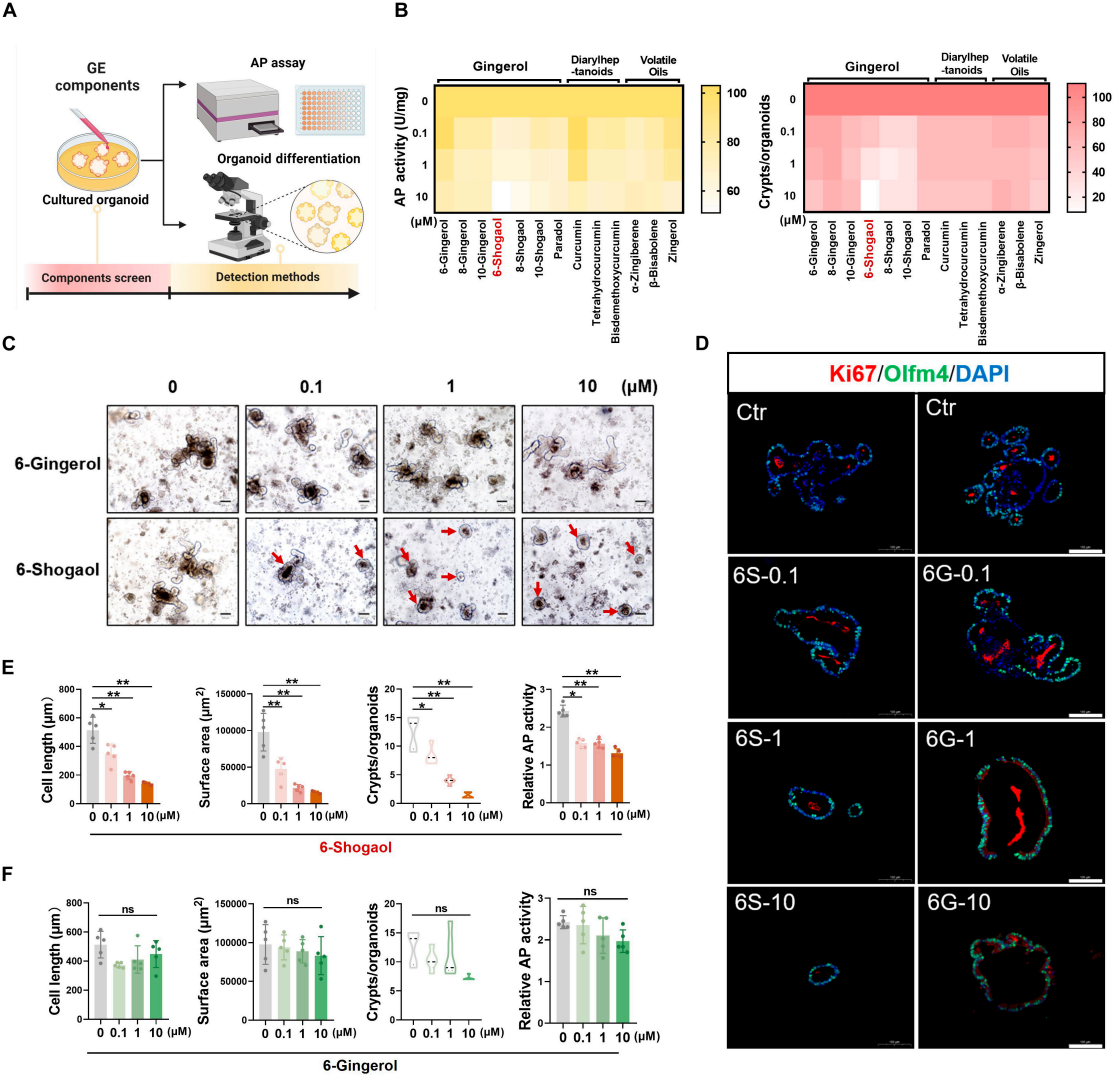
Intestinal organoid model screened-dietary ginger-derived 6-shogaol inhibits crypt stem cell differentiation. (A) Flow diagram of the screening of ginger toxic by-products using intestinal organoid differentiation model. Screening analysis of the main active components of ginger against (B) relative activity of AP and average number of crypt foci. (C) Toxicity analysis of ginger components, 6-shogaol, and 6-gingerol, using action and organoid differentiation. Scale bar (black), 100 μm (red arrows represent organoids with suppressed differentiation). (D) Immunofluorescence analysis of 6-shogaol and 6-gingerol toxicity in intestinal-like organs using Ki67 and Olfm4. Scale bar (white), 100 μm. (E and F) Analysis of maximum diameter length, mean surface area, mean crypt number, and AP enzymatic activity of 6-shogaol and 6-gingerol acting on intestinal organoids. Data are expressed as mean ± standard deviation (*n* = 6). *P < 0.05, **P < 0.01, as indicated.

Based on the effects of dietary ginger on the differentiation and regeneration of intestinal crypt stem cells (ICSCs), we constructed an intestinal organoid stem cell differentiation component screening model to analyze the main components of ginger that aggravate IBS (Fig. 2A). GE mainly comprises 3 types of active components: gingerol, diarylheptanoids, and volatile oil (Table S2) [14]. We integrated references to screen the organoid differentiation inhibition effect of the components with high ginger content based on the concentration of gastric and intestinal fluids [18,19]. We found that the main active ingredient, 6-gingerol, is the main product of gastric acid conversion in vivo and that 6-shogaol causes the most evident inhibition of the crypt differentiation of intestinal organoids. Compared with other gingerols, diarylheptanoids, and volatile oils, 6-shogaol significantly inhibited the average length, surface area, average crypt budding number, intestinal epithelial cell differentiation marker, and alkaline phosphatase (AP) activity index [20] of the organoids (Fig. 2B and Fig. S5A and B). Subsequently, we systematically compared the effects of 6-shogaol and its prototype component, 6-gingerol, on the differentiation of intestinal organoids. Compared to 6-gingerol, 6-shogaol significantly reduced the length of intestinal organoids and surface area of organoids, number of crypt buds, and activity of AP related to differentiation (Fig. 2C, E, and F). The immunofluorescence assay on small intestinal organoids determined that exposure markedly inhibited the expression of Ki67 and mucin 2 (MUC2), which is associated with proliferation and goblet cell differentiation (Fig. 2D and Fig. S6A).

### In vitro gastric acid and high-temperature stimulation to verify the in vivo transformation of toxic components in ginger

**Fig. 3.**
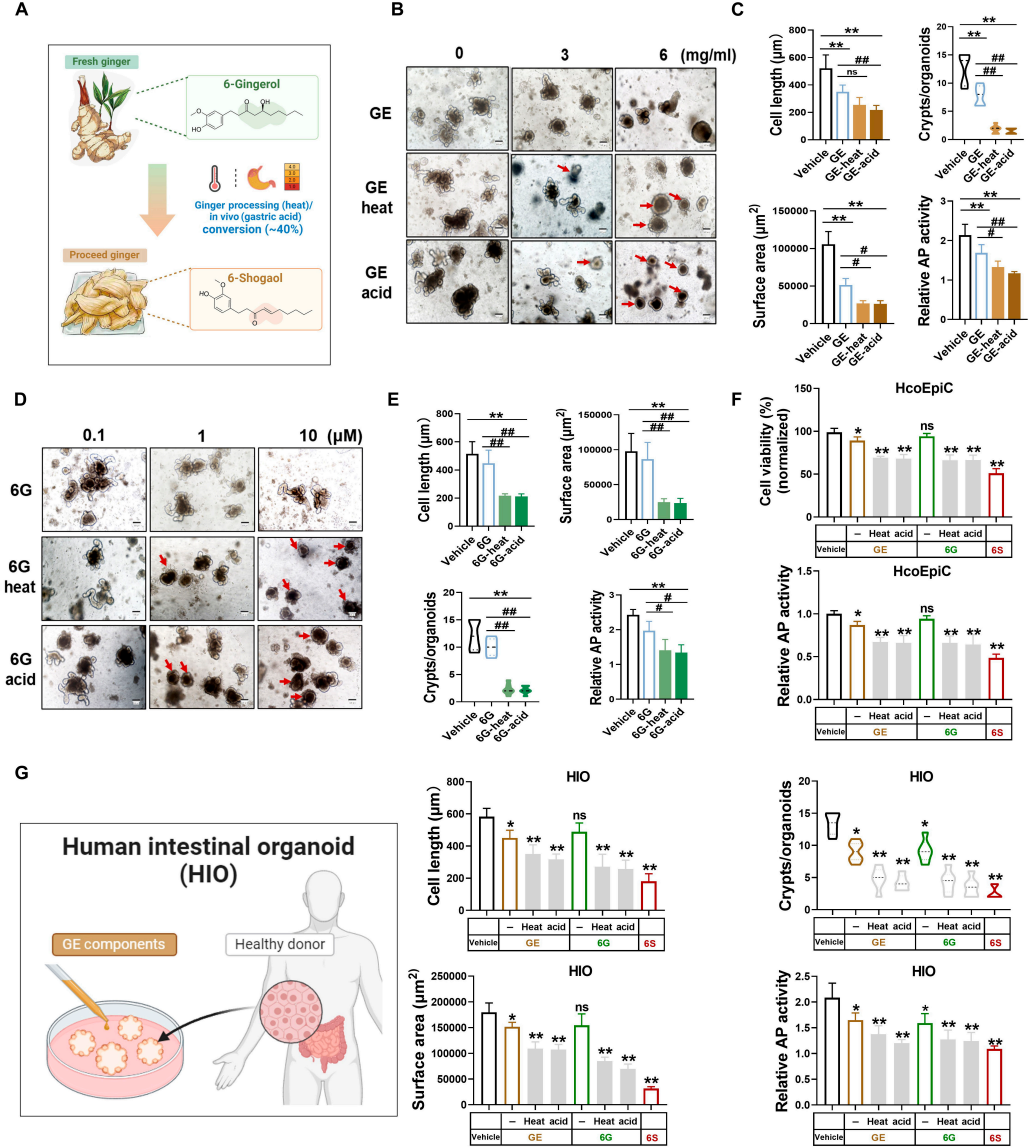
Gastric acid and high-temperature stimulation to verify the in vivo transformation of toxic components in ginger. (A) Thermal processing of ginger and the acidic environment of gastric juice promotes the conversion of 6-gingerol to 6-shogaol. (B) Comparative analysis of heat- and acid-treated organoid differentiation of GEs. Scale bar (black), 100 μm (red arrows represent organoids with suppressed differentiation). (C) Comparative analysis of the mean surface area, mean crypt number, maximum diameter length, and relative activity of AP in the heat- and acid-treated organoids of GEs. (D) Comparative analysis of organoid differentiation of heat and acid treatment of 6-gingerol. Scale bar (black), 100 μm (red arrows represent organoids with suppressed differentiation). (E) Comparative analysis of the average surface area, average number of crypt foci, maximum diameter length, and relative activity of AP in the heat- and acid-treated organoids of 6-gingerol. (F) Comparative analysis of the relative cell viability and activity of AP in the heat- and acid-treated human colonic epithelial cells (HcoEpiC) of GE, 6-gingerol, and 6-shogaol. (G) Comparative analysis of the average surface area, average number of crypt foci, maximum diameter length, and relative activity of AP in the heat- and acid-treated human intestinal organoids (HIO) of GE, 6-gingerol, and 6-shogaol. Data are expressed as mean ± standard deviation (*n* = 6). *P < 0.05, **P < 0.01, compared with the vehicle; ^#^ *P* < 0.05, ^##^ *P* < 0.01, compared with GE or 6-gingerol (6G) as indicated.

Gastric acid and heating conversions are the main processes involved in the preparation, processing, and digestion of ginger. Under acidic conditions, such as the presence of gastric acid, or heating conditions, such as processing, 6-gingerol is converted to 6-shogaol, with a conversion rate as high as 40% [21]. Accordingly, to verify the potential effect of ginger on the inhibition of intestinal recess differentiation in a real environment, we simulated the treatment of GE and its main component, 6-gingerol, in an in vitro gastric acid incubation and high-temperature processing (Fig. 3A). Based on the concentration of GE and its main components in the digestive fluid of mice [21], our findings indicate that the inhibitory effects of GE on the organoid surface area, crypt budding number, and differentiation-related AP activity, as well as the immunofluorescence intensity of MUC2, were markedly enhanced following treatment with high-temperature and under acidic conditions compared with the untreated group, as determined by the concentrations in gastric and intestinal fluids (Fig. 3B and C and Fig. S6B). Furthermore, a comparison of the main active ingredient, 6-gingerol, before and after treatment showed that high temperature and acid conversion enhanced the inhibition of organoid differentiation and regeneration (Fig. 3D and E and Fig. S6A). Furthermore, we applied human-derived primary intestinal epithelial cells (including ICSCs) and human small intestine organoids to verify the inhibitory effects of GE and 6-gingerol and its high temperature and acid conversion components on organoid differentiation and regeneration (Fig. 3F and G).

### In vivo IBS model validation confirms that ginger-derived 6-shogaol aggravates disease symptoms

**Fig. 4.**
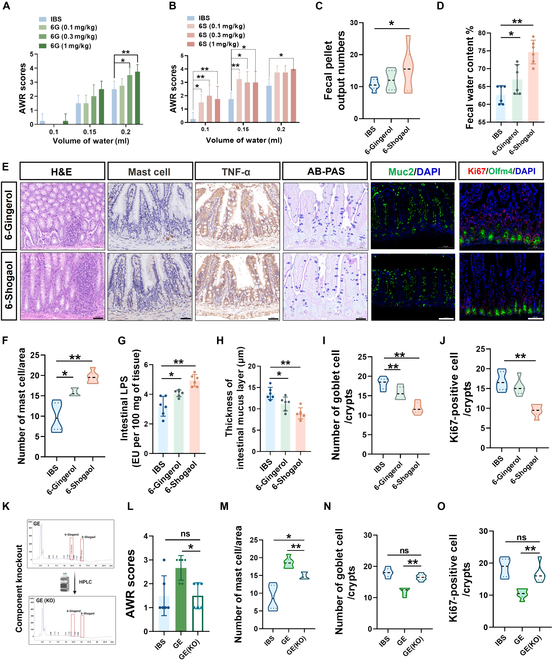
Experimental IBS model and component KO method to verify dietary ginger-derived 6-shogaol aggravates the disease index of IBS model mice. Application of the IBS model to verify the IBS-aggravating effect of ginger dietary ginger-derived 6-shogaol. Comparative analysis of (A and B) AWR score, (C) fecal water content (6-gingerol, 1 mg/kg; 6-shogaol, 1 mg/kg), and (D) fecal pellet count (6-gingerol, 1 mg/kg; 6-shogaol, 1 mg/kg) of 6-gingerol and 6-shogaol in IBS mice. (E) H&E and AB-PAS staining analysis of mouse intestinal tissues (ileum), immunohistochemical analysis of mast cell and TNF-α, and immunofluorescence analysis of mouse intestinal tissues for MUC2, Ki67, and Olfm4 in vehicle, IBS, 6-gingerol (1 mg/kg), and 6-shogaol (1 mg/kg) groups. Scale bar (black), 50 μm; scale bar (white), 100 μm. Comparative analysis of the intestinal section (ileum) (F) mast cell infiltration, (G) intestinal LPS level, (H) thickness of intestinal mucus layer, (I) mean number of goblet cells, (J) Ki67-positive cells per unit intestinal crypt in IBS, and 6-gingerol (1 mg/kg) and 6-shogaol (1 mg/kg) groups. (K) Component KO method to verify the IBS exacerbation components (6-gingerol and 6-shogaol) in ginger. Comparative analysis of (L) AWR score of GE (ginger raw materials 300 mg/kg) and GE (KO) (ginger raw materials 300 mg/kg) groups. Comparative analysis of intestinal section (ileum) (M) mast cell infiltration, mean number of (N) goblet cells, and (O) Ki67-positive cells per unit intestinal crypt in GE and GE (KO) groups. Data are expressed as mean ± standard deviation (*n* = 6). *P < 0.05, **P < 0.01, as indicated.

Based on 6-shogaol, a toxic component of ginger screened by our organoids in vitro, we used the IBS mouse model to verify the potential IBS-aggravating effect of 6-shogaol. Based on the content of 6-gingerol and 6-shogaol in ginger [21,22], we applied gavage experiment in mice with a conversion dose of 0.1 to 1 mg/kg 6-gingerol and 0.1 to 1 mg/kg 6-shogaol to verify the in vivo effect of ginger on exacerbating IBS (Fig. S7A). The results showed that >0.1 mg/kg 6-shogaol (equivalent to the content of 6-shogaol in 90 to 300 mg/kg ginger from in vivo study based on in vivo conversion data of ginger ingredients [21]) significantly increased the AWR score, number of defecation particles, and fecal water content of IBS mice in a dose-dependent manner (Fig. 4B to D). Additionally, in the 0.3 to 1 mg/kg 6-gingerol (equivalent to the content of 6-gingerol in 90 to 300 mg/kg ginger from in vivo study) group, a symptom phenotype that aggravated the mouse IBS disease index was also found under gastric acid transformation 6-shogaol conditions in vivo (Fig. 4A, C, and D). As a structural analog of 6-shogaol, capsaicin, which is the main component of pepper, also aggravates IBS symptoms; however, the toxic dose of 6-shogaol-induced toxicity was considerably lower than that of capsaicin (Fig. S8A to D). Further comparison of the results of the intestinal pathology and immunofluorescence data analysis revealed that similar to the results of the ginger treatment group, 6-gingerol and 6-shogaol increased inflammatory infiltration of the intestinal mucosa and inhibited the differentiation and regeneration of ICSCs. However, the inhibitory effect of 6-shogaol was stronger than that of 6-gingerol (Fig. 4E to J and Fig. S7B to D).

To study the main toxic role of 6-shogaol in dietary ginger, the component knockout method based on high-performance liquid chromatography (HPLC) was applied to remove the 6-gingerol and 6-shogaol in GE (Fig. 4K and Fig. S9A to D). The results showed that GE (6-gingerol and 6-shogaol knockout) [GE (KO)] treatment reversed the AWR score, number of defecation particles, and fecal water content index induced by GE treatment (Fig. 4L and Fig. S10A and B). Further GE (KO) treatment reversed the inflammatory infiltration and ICSC differentiation inhibition effect induced by GE treatment (Fig. 4M to O and Fig. S10C to H). Taken together, we find that 6-shogaol is the main toxic component in ginger. It inhibits crypt stem cell differentiation, exacerbates intestinal inflammation, and aggravates experimental IBS symptoms.

### 6-Shogaol inhibits ICSC differentiation by binding to eIF3A

**Fig. 5.**
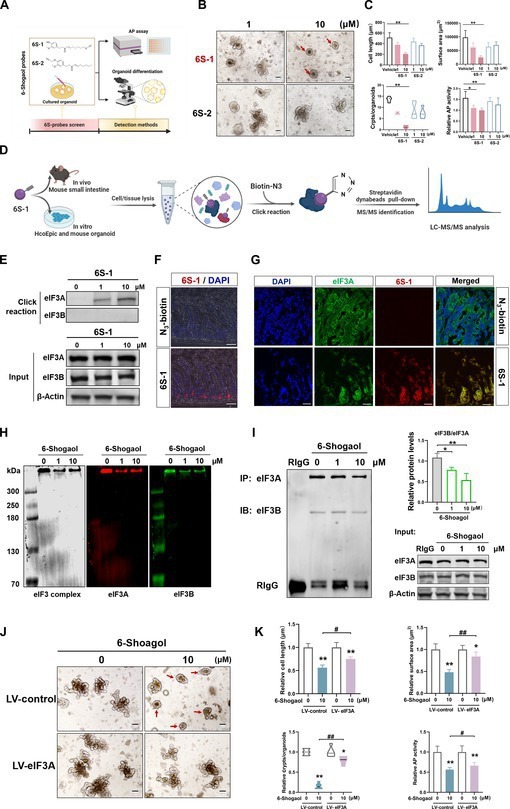
Ginger-derived 6-shogaol inhibits the differentiation of ICSCs by binding to eIF3A. (A) Synthesis of molecular probes containing alkyne groups at different sites of 6-shogaol. (B and C) Comparative analysis of the average surface area, average crypt number, maximum diameter length, and relative AP activity of 6-shogaol molecular probes of organoid. Scale bar (black), 100 μm (red arrows represent organoids with suppressed differentiation). (D) Click chemistry binding of 6-shogaol molecular probes to identify 6-shogaol binding target proteins. (E) Click chemistry-conjugated immunoblot analysis of the 6-shogaol binding target protein eIF3A. (F and G) Click chemistry-conjugated immunofluorescence analysis of 6-shogaol intestinal localization [scale bar (white), 100 μm] and binding target protein eIF3A [scale bar (white), 25 μm]. (H) Identification of the effect of 6-shogaol on the eIF3 translation initiation complex by nondeformable electrophoresis. (I) Immunoprecipitation to identify the effect of 6-shogaol on eIF3A-eIF3B interactions. (J and K) Exogenous lentivirus (LV)-eIF3A infection comparative analysis of 6-shogaol action on intestinal analogs with the mean surface area of analogs, mean number of crypt foci, maximum diameter, and AP activity. Scale bar (black), 100 μm (red arrows represent organoids with suppressed differentiation). Data are expressed as mean ± standard deviation (*n* = 6). *P < 0.05, **P < 0.01, compared to control. ^#^ *P* < 0.05, ^##^ *P* < 0.01, as indicated.

Based on the preliminary identification of the toxic substance, 6-shogaol, to further explore the specific mechanism of action, we synthesized alkynyl-containing molecular probes at the carbon chain (6S-1) and hydroxyl (6S-2) ends of 6-shogaol (Fig. 5A) and screened them using the intestinal organoid differentiation model. The inhibition of differentiation by the 6S-1 probe was similar to the toxic effect of 6-shogaol (Fig. 5B and C); therefore, 6S-1 was selected as the probe for subsequent molecular targeting experiments. Furthermore, we combined click chemistry and proteomic enrichment to identify the possible target proteins that 6-shogaol may bind to in both humans and mice (Fig. 5D and Fig. S11A and B). Based on the results of the enrichment analysis that evaluated the binding targets of 6-shogaol and its associated phenotypic inhibitory effects on ICSC differentiation, we determined that 6-shogaol specifically targets eukaryotic initiation factor 3A (eIF3A) protein (Fig. 5E), a member of the eIF3 family closely associated with the differentiation of intestinal stem cells [23]. Furthermore, immunofluorescent assays showed that 6-shogaol mainly accumulates in the ICSC region at the base of the intestinal crypt and colocalizes with eIF3A in mouse intestinal sections (Fig. 5F and G). While analyzing how 6-shogaol affects the biological function of eIF3A, the largest subunit of the eukaryotic translation initiation factor eIF3, we found that binding of 6-shogaol to eIF3A significantly impacts the formation of the translation initiation complex by inhibiting its interaction with other members of the eIF3 family (Fig. 5H and I). Verifying whether 6-shogaol inhibits the differentiation of ICSCs by combining eIF3A, increasing eIF3A expression effectively reversed the inhibition of the average length, surface area, average crypt budding number, and differentiation-related AP caused by 6-shogaol (Fig. 5J and K).

### 6-Shogaol blocks eIF3 transcription initiation complex by binding to eIF3A Cys^58^ site

**Fig. 6.**
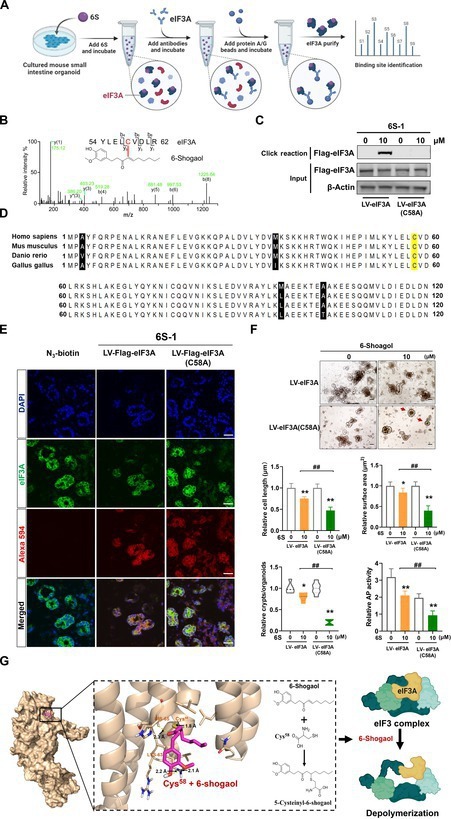
6-Shogaol blocks the eIF3 translation initiation complex by binding to the Cys^58^ site of eIF3A. (A) Flow chart of immunoprecipitation combined with mass spectrometric analysis of 6-shogaol binding sites. (B) Matrix-assisted laser desorption/ionization mass spectrometry showed the binding of 6-shogaol to peptides containing eIF3A-Cys^58^. (C) Effect of exogenous infection with LV-eIF3A (C58A) lentivirus and 6-shogaol on eIF3A-eIF3B interbinding. (D) Amino acid sequence alignments of full-length eIF3A. Yellow highlight, conserved cysteine (Cys^58^) in eIF3A protein. (E) Exogenous infection with LV-eIF3A (C58A) lentivirus and click chemistry combined with immunofluorescence analysis of 6-shogaol binding target protein eIF3A site specificity. Scale bar (white), 25 μm. (F) Exogenous infection with LV-eIF3A mutation (C58A) and comparative analysis of the mean surface area, mean number of crypt foci, maximum diameter, and relative AP activity of organoids. Scale bar (black), 100 μm (red arrows represent organoids with suppressed differentiation). (G) Crystal structure of eIF3A in complex with 6-shogaol at Cys^58^ causes the depolymerization of eIF3 complex. Data are expressed as mean ± standard deviation (*n* = 6). *P < 0.05, **P < 0.01, compared to control. ^#^ *P* < 0.05, ^##^ *P* < 0.01, as indicated.

We further used immunoprecipitation to enrich eIF3A binding to 6-shogaol (Fig. 6A) and mass spectrometry to identify its possible binding sites and found that 6-shogaol specifically binds to the cysteine-58 (Cys^58^) site of eIF3A (Fig. 6B). The Cys^58^ site of eIF3A bound to 6-shogaol was highly conserved in humans and mice and reacted with the sulfhydryl group of cysteine to produce 5-cysteinyl-6-shogaol (Fig. 6D). Furthermore, we constructed an exogenous Cys^58^ site-mutated eIF3A for validation and found that the exogenous mutated eIF3A did not effectively bind to 6-shogaol (Fig. 6C) and lacked colocalization with 6-shogaol (Fig. 6E). The introduction of exogenous Cys^58^ site-mutated eIF3A did not significantly affect the inhibitory effects of 6-shogaol on the mean length, surface area, mean crypt emergence number, or differentiation-related AP enzymes in intestinal-like organs (Fig. 6F and G).

**Fig. 7.**
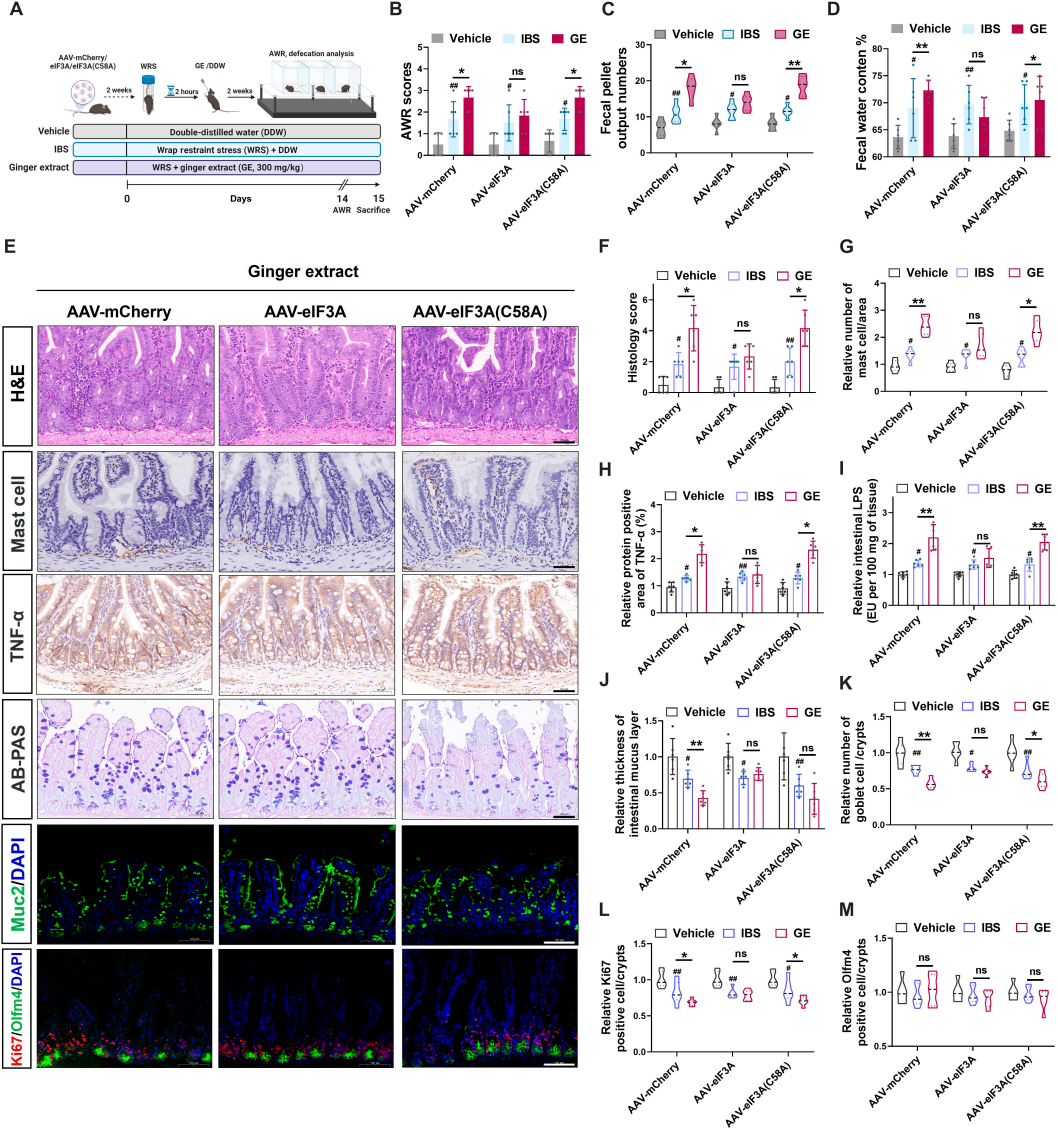
Overexpression of eIF3A by adenovirus infection reverses the adverse reactions of dietary ginger on IBS. (A) Application of AAV-mCherry, AAV-eIF3A, and AAV-eIF3A (C58A) to verify the IBS-aggravating mechanism of ginger. Comparative analysis of (B) AWR score, (C) fecal water content, and (D) fecal pellet count of AAV-mCherry-, AAV-eIF3A-, and AAV-eIF3A (C58A)-infected vehicle, IBS, and GE (300 mg/kg) groups. (E) H&E and AB-PAS staining analysis of mouse intestinal tissues (ileum), immunohistochemical analysis of mast cell and TNF-α, and immunofluorescence analysis of mouse intestinal tissues for MUC2, Ki67, and Olfm4 in AAV-mCherry-, AAV-eIF3A-, and AAV-eIF3A (C58A)-infected GE (300 mg/kg) group. Scale bar (black), 50 μm; scale bar (white), 100 μm. Comparative analysis of intestinal section (ileum): (F) histology score; (G) mast cell infiltration; (H) TNF-α abundance; (I) intestinal LPS level; (J) thickness of intestinal mucus layer; and mean number of (K) goblet cells, (L) Ki67, and (M) Olfm4-positive cells per unit intestinal crypt of AAV-mCherry-, AAV-eIF3A-, and AAV-eIF3A (C58A)-infected vehicle, IBS, and GE (300 mg/kg) groups. Data are expressed as mean ± standard deviation (*n* = 6). *P < 0.05, **P < 0.01, as indicated.

Furthermore, we applied adeno-associated virus (AAV)-mCherry, AAV-eIF3A, and AAV-eIF3A (C58A) to verify the mechanism of ginger-induced IBS aggravation. Compared with AAV-mCherry, AAV-eIF3A reversed the AWR score, number of defecation particles, and fecal water content index induced by GE treatment; however, AAV-eIF3A (C58A) did not show the relative reversed effect (Fig. 7A to D). Similarly, AAV-eIF3A infection reversed the inflammatory infiltration and inhibited crypt stem cell differentiation induced by ginger treatment; however, AAV-eIF3A (C58A) did not induce a relatively reversed effect (Fig. 7E to M), given the mutation in the binding site for 6-shogaol, making it ineffective for inhibition of eIF3A by 6-shogaol. Taken together, ginger-derived 6-shogaol bound to the eIF3A at Cys^58^ and inhibited the formation of the transcription initiation complex eIF3, thereby inhibiting crypt stem cell differentiation, exacerbating intestinal inflammation, and aggravating experimental IBS symptoms.

## Discussion

**Fig. 8.**
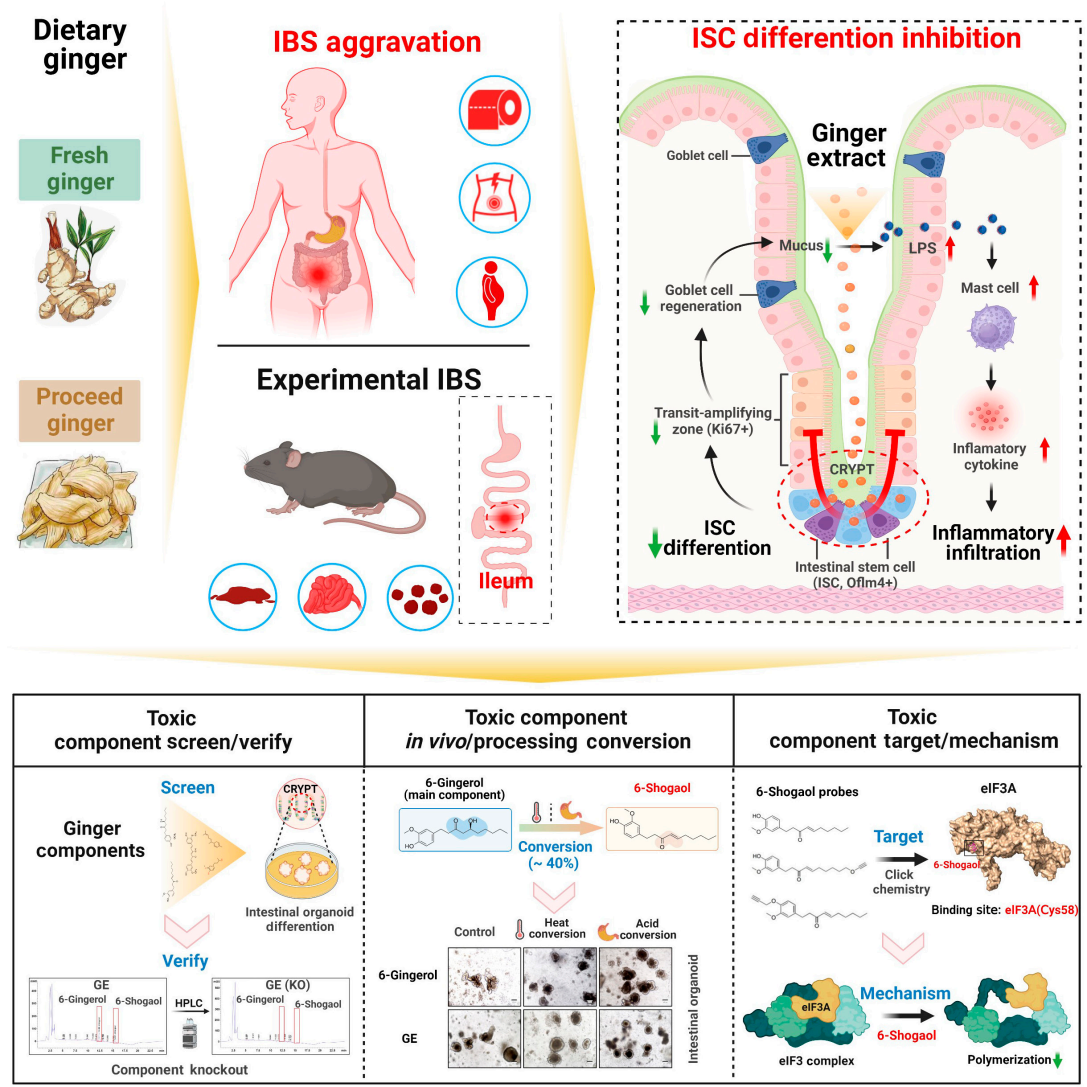
Dietary ginger-derived 6-shogaol exacerbates intestinal inflammation by inhibiting crypt stem cell differentiation in the experimental IBS model. Dietary ginger-derived 6-shogaol, a main component derived from 6-gingerol (ginger main ingredients) in vivo, through gastric acid and processing (heat) transformation, inhibited the eIF3 translation initiation complex formation by covalently binding to the Cys^58^ site of eIF3A, a key factor regulating ICSC differentiation, and further reduced the goblet cell number and its secretory function-related mucus layer thickness, resulting in increased LPS infiltration and low-grade inflammation (due to mast cell and inflammatory cytokines) in the ileum crypts, finally exacerbating symptoms of IBS in mouse model. Created using BioRender: https://BioRender.com/i43i875.

IBS is a chronic gastrointestinal disease that often requires considerable dietary management [4,24]. Although mainstream dietary interventions, such as low FODMAPs, can alleviate the symptoms of IBS and are considered low risk, safety concerns about such diets require further study [2,3]. Here, we demonstrated that the daily intake of 90 to 300 mg/kg of ginger (equivalent to a human daily dose of 0.6 to 2 g per person) may pose a risk of idiosyncratic toxicity [25–27], exacerbating symptoms in specific IBS populations. We found that 6-shogaol in ginger exacerbated intestinal symptoms by inhibiting ICSC differentiation in experimental IBS. Our study reveals a novel mechanism underlying the impact of ginger on IBS symptoms, potentially influencing the direction of clinical use of ginger in treating IBS (Fig. 8). Ginger has long been considered a safe edible and medicinal homolog plant [12], due to which careful attention has not been paid to the side effects associated with its intake. The IBS incidence rate associated with ginger top consumption countries is high, particularly in some Asian and African countries with no-FODMAP diets [1,28]. This indicates a subtle relationship between ginger consumption and IBS incidence. However, there is considerable confusion for patients with IBS seeking dietary therapy, and ginger is not included in the list of dietary items excluded for IBS. Therefore, improving research on the use of ginger in IBS, correcting misconceptions about the current application of ginger in gastrointestinal diseases, and addressing population-specific issues is essential to ensure the safe clinical use of ginger.

In susceptible individuals with IBS, infection or consumption of certain foods increases intestinal permeability by altering epithelial cell function, and further localized inflammation develops with a subsequent influx of inflammatory cells [29–34]. Our study demonstrated that the intake of ginger increased mast cell infiltration and inflammatory factor expression in the intestinal mucosa of an IBS model, indicating that the IBS symptoms aggravated by ginger were related to the level of advanced inflammation. The intestinal epithelial barrier is a key factor controlling food-induced intestinal inflammation [35]. Furthermore, ginger intake inhibited the renewal of intestinal goblet cells by inhibiting the differentiation of ICSCs, ultimately decreasing mucus thickness and LPS, low-grade inflammatory infiltration of the intestine, and the exacerbation of IBS. To the best of our knowledge, this is the first study to propose that food affects ICSC differentiation, causing dysfunction of the intestinal mucus barrier and inducing increased inflammatory infiltration of the intestinal mucosa, ultimately exacerbating the symptoms of IBS. Previous findings on the components of food-induced IBS were from clinical trials and screening based on IBS animal models. However, an efficient screening model that connects IBS clinical findings in humans with food-induced components remains lacking. Our study was based on the inhibitory effect of ginger on the differentiation of ICSCs, and we developed a systematic screening strategy for ginger-induced components in the gut. Organoid-derived intestinal epithelial cells generated from intestinal organoids possess physiological properties similar to those of the intestinal epithelium and can serve as tools for enhanced prediction of biological activity in humans. Unlike monolayer-cultured intestinal epithelial cells, organoid-derived intestinal epithelial cells are a suitable model for preclinical toxicology and pharmacokinetic studies [36,37]. However, they are rarely applied in screening ingredients for food-induced IBS, although intestinal organoids are an excellent tool for studying the differentiation and barrier functions of intestinal stem cells related to IBS. Further screening of food-inducing components using intestinal organoids revealed that the main active ingredient in ginger is 6-gingerol, and its by-product, 6-shogaol, significantly inhibited the differentiation of intestinal organs and aggravated IBS symptoms.

This contradicts recent research suggesting that ginger alleviates IBS through 6-gingerol inhibition of intestinal inflammatory factor expression. This dichotomy is owing to the omission of the conversion of 6-gingerol to 6-shogaol in the digestive system [38]. During the heating and gastric acid digestion processes (under pH < 4), 6-gingerol is largely converted to 6-shogaol (conversion rate of >40%) in fresh ginger [21]. Although hardly soluble, 6-shogaol shows good stability in the simulated intestinal fluid within 2 h, with a maximum solubility of 10 μM [18,19]. Thus, a large amount of 6-shogaol can accumulate under heating and gastric acid conditions, causing inhibition of ICSC differentiation. Furthermore, in the colon, 6-shogaol is extensively converted to 6-paradol by the gut microbiota [39]. This indicates that 6-shogaol mainly causes pathological damage to the small intestine. Notably, capsaicin, a structural analog of 6-shogaol, exhibits potential toxicity against chronic inflammation and cancer [40]. Furthermore, the toxic dose of 6-shogaol was lower than that of capsaicin. This may be because 6-shogaol has a greater direct effect on the differentiation of ICSCs than capsaicin analogs found in spicy food sources, which bind to transitional receptor potential vanilloid subtype 1 in intestinal mucosal neurons [41]. Decreased eIF3A expression may be a prerequisite for intestinal epithelial cell differentiation [23,42]. 6-Shogaol binds at the N-terminal domain Ser^51^ site of eIF2A [43]; however, in this study, 6-shogaol covalently bound to the Cys^58^ site of eIF3A, consequently affecting the polymerization of translation initiation complexes of eIF3, with significant effects on ICSC–epithelial cell differentiation. The ultimate toxicant forms irreversible complexes with body macromolecules, such as proteins, which alter the function of biological macromolecules in the body and produce toxicity [44]. In this study, the covalent binding of 6-shogaol to eIF3A greatly affected the differentiation of ICSCs, ultimately leading to exacerbated symptoms of IBS.

We also used enteritis models, which showed that ginger exacerbated the disease index and chronic inflammatory infiltration of the intestine in mice models. More intestinal disease models are needed to expand the contraindications of ginger. Meanwhile, organoid platforms for detecting food enterotoxicity require further development. Furthermore, plant derived components can affect the progression of intestinal diseases by regulating the gut microbiota [45,46]. Our study further implicates the involvement of gut microbiota in ginger-induced toxicity (data not shown). A comprehensive examination of the gut microbiota associated with IBS, the degranulation of intestinal mast cells, and the biotransformation of ginger constituents by the microbiota is essential for elucidating the toxicological effects of ginger on IBS. The existing clinical trials on ginger are limited in scale; therefore, the specific effects on IBS and other gastrointestinal conditions require further investigation with larger sample sizes and advanced statistical methods, such as Mendelian randomization analysis. Future studies on ginger-exacerbated IBS should be conducted with robust clinical endpoints, standard comparators, and diverse populations. Our study showed that understanding the exacerbating effect of ginger on IBS is essential for the rational, clinical, and safe use of ginger in IBS, providing new approaches for the safety evaluation of food-induced IBS.

## Materials and Methods

### Ginger preparation

Ginger rhizomes were purchased from a herbal medicine market in Nanjing, China, and washed with deionized water thrice at 20 °C. Based on the consumption, medicinal dosage, and habits of ginger consumption in daily life [6,14], fresh ginger juice (GE) and air-dried ginger rhizomes samples (GP) were prepared to be used in animal and organoid models. For GE, fresh ginger was ground with a food processor and preserved at 4 °C. The juice was then collected after the removal of cellular debris by centrifugation at 5,000*g* for 5 min at room temperature. Finally, 1.5 g/ml ginger juice was prepared at room temperature. The liquid was filtered and used as GE for animal and organoid treatment. The property of the extract was profiled using HPLC analysis. For GP, air-dried ginger was ground into a 200-mesh powder. The fine powder was suspended in distilled water and was orally administered for the animal study.

### Establishment of IBS model

C57BL/6 male mice (6 to 8 weeks old), weighing 20 to 22 g, were purchased from Beijing Vital River Laboratory Animal Technology Co. Ltd. (China). The stress-IBS model was established and initiated by repeated wrap restraining stress. Briefly, the mice were tightly restrained in the 50-ml tubes with a hole for air. This restraint procedure minimized the space around the animal, prevented turning, and provided a strong, stressful stimulus without being harmful in the long run. Restraint sessions lasted for 2 h in the morning (09:00 AM to 12:00 PM) and were repeated daily for 2 consecutive weeks [47,48]. For ginger-exacerbated IBS studies, 30 to 300 mg/kg (original fresh ginger quantity) GE and GP (equivalent to a daily human dose of 0.2 to 2 g per person based on ginger daily consumption data, the dosage used in clinical trials, and equivalent dose ratio converted from body surface area [6,14]) were administered daily by gavage to the mice immediately after the restrain stress procedure for 2 weeks for the IBS-wrap restrain stress model (Fig. 1A). For ginger-derived compound exacerbated IBS studies, 0.1 to 1 mg/kg 6-gingerol and 0.1 to 1 mg/kg 6-shogaol (equivalent to 30 to 300 mg/kg GE based on the content of these 2 components in ginger, internal digestive fluid conversion rate [21,22,49], and experimental control requirements) and 0.1 to 10 mg/kg capsaicin (based on the clinical trial dosage of capsaicin [40,50] and comparison requirements with the 6-shogaol experiment) were daily gavage to the mice immediately after the restrain stress for 2 weeks for the IBS-wrap restraining stress model (Figs. S6A and S7A).

After treatment, the fecal water content, fecal pellet output, and visceral sensitivity were measured in each group on day 15 [51]. The AWR was semiquantitatively scored as previously described [52]. Two other models of functional gastrointestinal diseases related to inflammation were applied to expand the validation of ginger-induced intestinal adverse reactions, and the specific experimental steps are reflected in the supplementary materials. All procedures involving animals were approved by the Institutional Animal Care and Use Committee of the Jiangsu Province Institute of Traditional Chinese Medicine (ethical approval number: AEWC-20200520-108), and procedural details were drafted according to the ARRIVE guidelines. The experiments were performed in accordance with the guidelines of the National Institutes of Health.

### Crypt isolation and organoid culture

Small intestinal tissues were collected from the mice and human donors. The human organoid line in use was derived by intestinal endoscopic biopsy from the ileum. Informed consent was obtained from all patients, and the study was approved by the ethics committee of the Jiangsu Province Institute of Traditional Chinese Medicine (ethical approval number: 2022-LWKYS-021). This study is compliant with all relevant ethical regulations regarding research involving human participants. Intestinal crypts were isolated using the Gentle Cell Dissociation Reagent (StemCell Technologies, MA, USA). The tissues were incubated with 0.1% bovine serum albumin, and the cell suspension was passed through a 70-μm cell strainer. Isolated crypts were observed under a microscope (CKX41; Olympus, Tokyo, Japan). The crypts were mixed with Matrigel (Corning, USA) and IntestiCult Organoid Growth Medium (Mouse) (StemCell Technologies) at a ratio of 1:1, and 50 μl of the suspended crypts was placed in 24-well plates. After polymerization by incubating at 37 °C for 10 min, 600 μl of IntestiCult Organoid Growth Medium was added, and the plate was placed in a humidified incubator (5% CO_2_) at 37 °C. The culture medium was replaced once every 2 days. Photographs were taken using an inverted fluorescence microscope. The length of the organoids, lumen–cell length ratio, number of buds, and crypt and villus domains were measured using ImageJ software (V1.8.0; National Institutes of Health, Bethesda, MD, USA) [53].

### Statistical analysis

Data are expressed as mean ± standard deviation. Statistical analyses and graphs were generated using GraphPad Prism software (version 7.0, GraphPad Software Inc., La Jolla, CA, USA). Correlation between normally distributed data was analyzed using the Pearson or Spearman analysis. Statistical differences between the 2 groups were analyzed using an unpaired 2-tailed Student’s *t* test. For multiple groups, statistical differences were analyzed using a one-way analysis of variance. Differences between means were considered statistically significant at *P* < 0.05. Other experimental methods are listed in the Supplementary Materials.

## Data Availability

All data relevant to the study are included in the article or uploaded as information.
