# Supplementary material for: 6-Shogaol Derived from Ginger Inhibits Intestinal Crypt Stem Cell Differentiation and Contributes to Irritable Bowel Syndrome Risk
Source: Research (Wash D C). 2024 Nov 7;7:0524. doi: 10.34133/research.0524 (PMC11542252; doi:10.34133/research.0524)
Supplement: Supplementary 1 — Figs. S1 to S11 Tables S1 and S2 [file research.0524.f1.docx]

**Supplementary Information**

6-Shogaol Derived from Ginger Inhibits Intestinal Crypt Stem Cell Differentiation and Contributes to Irritable Bowel Syndrome Risk

Bing Zhao^1,2†^, Juan Ye^1,2†^, Wenjing Zhao^1,2^, Xinyu Liu^1,2^, Hongli Lan^1,2^, Jinbing Sun^3^, Jiao Chen^1,2^, Xueting Cai^1,2^, Qingyun Wei^1,2^, Qian Zhou^1,2^, Zhengwei Zhang^1,2^, Yuze Wu^1,2^, Yang Yang^1,2*^, and Peng Cao^1,2*^

^1^China National Key Laboratory on Technologies for Chinese Medicine Pharmaceutical Process Control and Intelligent Manufacture, Nanjing University of Chinese Medicine, Nanjing, China

^2^Jiangsu Provincial Medical Innovation Center, Affiliated Hospital of Integrated Traditional Chinese and Western Medicine, Nanjing University of Chinese Medicine, Nanjing, China

^3^Department of General Surgery, Changshu No. 1 People’s Hospital, Affiliated Changshu Hospital of Soochow University, Changshu, China

^*^Address correspondence to: Yang Yang; [young1570@126.com](mailto:young1570@126.com) and Peng Cao; [cao_peng@njucm.edu.cn](mailto:cao_peng@njucm.edu.cn)

†These authors contributed equally to this work and share first authorship.

**Supplementary Tables**

**Table S1.** **Correlation analysis of the global irritable bowel syndrome prevalence,** **per capita consumption of ginger, and IBS-related foods under Manning and Rome I–IV criteria**

| **IBS diagnostic criteria** | | | **Dietary food (g/per person day)** | | | | |
| --- | --- | --- | --- | --- | --- | --- | --- |
| **Region** | | **Manning criteria (%)** | **Ginger** | **Wheat** | **Garlic** | **Milk** | **Processed meats** |
| United States | | 16.0 | 0.39 | 304.63 | 2.28 | 898.58 | 72.34 |
| United Kingdom | | 19.0 | 0.78 | 589.55 | 1.34 | 712.27 | 165.99 |
| Sweden | | 19.0 | 0.22 | 569.53 | 0.82 | 1266.39 | 140.38 |
| Netherlands | | 6.0 | 1.07 | 846.73 | 0.72 | 2358.57 | 100.74 |
| France | | 2.0 | 0.12 | 866.20 | 1.35 | 1507.45 | 142.93 |
| Finland | | 10.0 | 0.09 | 373.39 | 0.48 | 1809.85 | 280.71 |
| Iceland | | 31.0 | 0.69 | 332.06 | 0.89 | 1575.90 | 92.63 |
| Singapore | | 5.0 | 3.55 | 114.13 | 5.94 | 29.46 | 27.42 |
| South Korea | | 15.0 | 0.65 | 227.33 | 19.36 | 203.32 | 10.26 |
| China | | 10.0 | 0.13 | 228.85 | 32.44 | 73.83 | 38.98 |
| Australia | | 14.0 | 0.10 | 845.48 | 1.37 | 1524.48 | 152.25 |
| **Correlation coefficient** | | | -0.23 | -0.2 | -0.12 | 0.03 | 0.02 |
| ***p*-value** | | | 0.52 | 0.57 | 0.73 | 0.93 | 0.96 |
|  | | |  |  |  |  |  |
| **IBS diagnostic criteria** | | **Dietary food (g/per person day)** | | | | |  |
| **Region** | **Rome I criteria (%)** | **Ginger** | **Wheat** | **Garlic** | **Milk** | **Processed meats** |  |
| United States | | 9.0 | 0.39 | 304.63 | 2.28 | 898.58 | 72.34 |
| Canada | | 13.0 | 0.75 | 469.11 | 1.28 | 873.44 | 142.82 |
| United Kingdom | | 10.0 | 0.78 | 589.55 | 1.34 | 712.27 | 165.99 |
| Sweden | | 14.0 | 0.22 | 569.53 | 0.82 | 1266.39 | 140.38 |
| France | | 3.0 | 0.12 | 866.20 | 1.35 | 1507.45 | 142.93 |
| Finland | | 6.0 | 0.09 | 373.39 | 0.48 | 1809.85 | 280.71 |
| Russia | | 19.0 | 0.05 | 750.46 | 5.29 | 742.22 | 123.45 |
| Germany | | 6.0 | 0.24 | 666.76 | 0.56 | 1384.72 | 194.73 |
| Spain | | 14.0 | 0.09 | 605.95 | 5.80 | 584.89 | 118.25 |
| Italy | | 7.0 | 0.07 | 608.80 | 2.08 | 766.99 | 130.10 |
| Romania | | 14.0 | 0.01 | 574.66 | 8.74 | 707.80 | 77.83 |
| Croatia | | 28.0 | 0.03 | 378.31 | 4.35 | 593.78 | 107.01 |
| Bangladesh | | 8.0 | 1.89 | 56.41 | 4.11 | 72.50 | 51.07 |
| Singapore | | 10.0 | 3.55 | 114.13 | 5.94 | 29.46 | 27.42 |
| South Korea | | 9.0 | 0.65 | 227.33 | 19.36 | 203.32 | 10.26 |
| China | | 2.4 | 0.13 | 228.85 | 32.44 | 73.83 | 38.98 |
| Australia | | 8.0 | 0.10 | 845.48 | 1.37 | 1524.48 | 152.25 |
| **Correlation coefficient** | | | -0.1 | 0.03 | -0.2 | -0.16 | -0.07 |
| ***p-*value** | | | 0.69 | 0.9 | 0.43 | 0.54 | 0.78 |
|  | | |  |  |  |  |  |
| **IBS diagnostic criteria** | | | **Dietary food (g/per person day)** | | | | |
| **Region** | | **Rome II criteria (%)** | **Ginger** | **Wheat** | **Garlic** | **Milk** | **Processed meats** |
| United States | | 7.0 | 0.39 | 304.63 | 2.28 | 898.58 | 72.34 |
| Canada | | 18.0 | 0.75 | 469.11 | 1.28 | 873.44 | 142.82 |
| United Kingdom | | 10.0 | 0.78 | 589.55 | 1.34 | 712.27 | 165.99 |
| Sweden | | 13.0 | 0.22 | 569.53 | 0.82 | 1266.39 | 140.38 |
| France | | 2.6 | 0.12 | 866.20 | 1.35 | 1507.45 | 142.93 |
| Finland | | 5.0 | 0.09 | 373.39 | 0.48 | 1809.85 | 280.71 |
| Germany | | 12.0 | 0.24 | 666.76 | 0.56 | 1384.72 | 194.73 |
| Italy | | 7.0 | 0.07 | 608.80 | 2.08 | 766.99 | 130.10 |
| Turkey | | 11.0 | 0.02 | 118.78 | 3.97 | 1.25 | 20.42 |
| Iran | | 9.0 | 0.04 | 439.89 | 2.17 | 436.07 | 82.30 |
| Nigeria | | 32.0 | 4.12 | 52.39 | 0.01 | 12.29 | 23.88 |
| Bangladesh | | 8.0 | 1.89 | 56.41 | 4.11 | 72.50 | 51.07 |
| Pakistan | | 28.0 | 0.92 | 392.51 | 2.04 | 566.97 | 53.13 |
| Singapore | | 9.0 | 3.55 | 114.13 | 5.94 | 29.46 | 27.42 |
| South Korea | | 6.0 | 0.65 | 227.33 | 19.36 | 203.32 | 10.26 |
| China | | 5.0 | 0.13 | 228.85 | 32.44 | 73.83 | 38.98 |
| Japan | | 6.0 | 2.65 | 137.96 | 0.85 | 202.49 | 25.14 |
| Malaysia | | 16.0 | 4.26 | 117.59 | 8.70 | 7.78 | 102.92 |
| Brazil | | 17.0 | 0.48 | 141.86 | 3.58 | 455.98 | 81.54 |
| Australia | | 7.0 | 0.10 | 845.48 | 1.37 | 1524.48 | 152.25 |
| Israel | | 3.0 | 0.19 | 655.26 | 3.83 | 556.21 | 140.53 |
| **Correlation coefficient** | | | 0.45***** | -0.32 | -0.24 | -0.26 | -0.24 |
| ***p*-value** | | | 0.04 | 0.15 | 0.28 | 0.26 | 0.3 |
|  | | |  |  |  |  |  |
| **IBS diagnostic criteria** | | | **Dietary food (g/per person day)** | | | | |
| **Region** | | **Rome III criteria (%)** | **Ginger** | **Wheat** | **Garlic** | **Milk** | **Processed meats** |
| Bangladesh | | 10.7 | 2.42 | 85.83 | 6.29 | 70.79 | 58.73 |
| China | | 3.8 | 0.17 | 248.92 | 34.44 | 74.55 | 54.71 |
| Ghana | | 0.4 | 0.02 | 54.11 | 0.34 | 4.66 | 3.44 |
| Indonesia | | 6.2 | 1.91 | 82.49 | 5.28 | 16.45 | 27.90 |
| Malaysia | | 3.9 | 4.72 | 114.46 | 9.84 | 7.48 | 126.50 |
| Nigeria | | 5.1 | 6.74 | 41.17 | 0.05 | 11.49 | 24.07 |
| Turkey | | 0.9 | 0.07 | 171.71 | 3.14 | 0.94 | 22.90 |
| **Correlation coefficient** | | | 0.37 | -0.22 | 0.04 | 0.62 | 0.28 |
| ***p*-value** | | | 0.41 | 0.64 | 0.94 | 0.14 | 0.54 |
|  | | |  |  |  |  |  |
| **IBS diagnostic criteria** | | | **Dietary food (g/per person day)** | | | | |
| **Region** | | **Rome Ⅳ criteria (%)** | **Ginger** | **Wheat** | **Garlic** | **Milk** | **Processed meats** |
| Bangladesh | | 4.6 | 2.42 | 85.83 | 6.29 | 70.79 | 58.73 |
| China | | 1.4 | 0.17 | 248.92 | 34.44 | 74.55 | 54.71 |
| Ghana | | 0.3 | 0.02 | 54.11 | 0.34 | 4.66 | 3.44 |
| Indonesia | | 3.5 | 1.91 | 82.49 | 5.28 | 16.45 | 27.90 |
| Iran | | 2.1 | 0.08 | 459.20 | 2.01 | 417.93 | 94.91 |
| Nigeria | | 2.7 | 6.74 | 41.17 | 0.05 | 11.49 | 24.07 |
| Turkey | | 0.4 | 0.07 | 171.71 | 3.14 | 0.94 | 22.90 |
| **Correlation coefficient** | | | 0.86***** | -0.11 | 0.29 | 0.43 | 0.61 |
| ***p*-value** | | | 0.02 | 0.84 | 0.56 | 0.35 | 0.17 |

* Indicates a significant correlation at the 0.05 level (two-tail).

The global prevalence data on diagnosing irritable bowel syndrome (IBS) using Manning (n = 11, United States, United Kingdom, Sweden, Netherlands, France, Finland, Iceland, Singapore, South Korea, China, and Australia), Rome I (n = 17, United States, Canada, United Kingdom, Sweden, France, Finland, Russia, Germany, Spain, Italy, Romania, Croatia, Bangladesh, Singapore, South Korea, China, and Australia), and Rome II criteria (n=21, United States, Canada, United Kingdom, Sweden, France, Finland, Germany, Italy, Turkey, Iran, Nigeria, Bangladesh, Pakistan, Singapore, South Korea, China, Japan, Malaysia, Brazil, Australia, and Israel) were collected from ref 1 [1], and the data on relative per capita consumption of food between 2007 and 2012 were collected from Indexbox [2]. The global prevalence data on diagnosing IBS using the Rome III (n = 7, Bangladesh, China, Ghana, Indonesia, Iran, Nigeria, and Turkey) and Rome IV criteria (n = 7, Bangladesh, China, Ghana, Indonesia, Malaysia, Nigeria, and Turkey) was collected from ref 3 [3], and the data on relative per capita food consumption between 2007 and 2020 were collected from Indexbox [2].

**Table S2. Main active ingredients in ginger**

| **Constituent** | **Essential component** | **Mass fraction%** |
| --- | --- | --- |
| **Gingerol** | 6-Gingerol | 0.067–0.391 |
|  | 8-Gingerol | 0.008–0.047 |
|  | 10-Gingerol | 0.010–0.084 |
|  | 6-Shogaol | 0.009–0.101 |
|  | 8-Shogaol | 0.009–0.089 |
|  | 10-Shogaol | 0.010–0.127 |
| **Diarylheptanoids** | Curcumin | 0.060–0.340 |
|  | Tetrahydrocurcumin | 0.030–0.275 |
|  | Bisdemethoxycurcumin | 0–0.007 |
| **Volatile oils** | α-zingiberene | 0.138–0.592 |
|  | β-bisabolene | 0.026–0.100 |
|  | Zingerol | 0.0076–0.010 |

**Supplementary** **Figures**


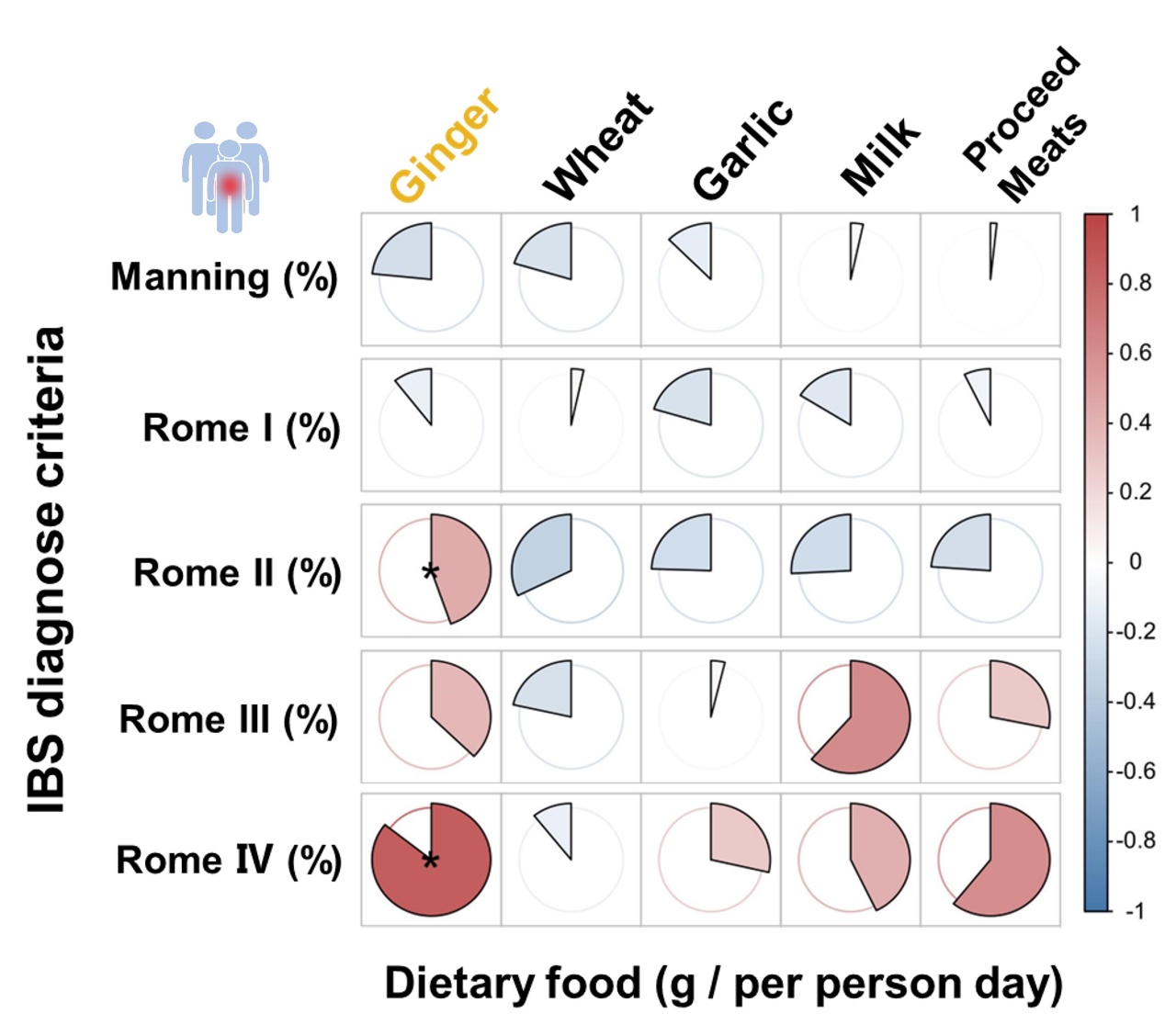


**Fig. S1.** **Correlation analysis of \global IBS prevalence, consumption of ginger, and IBS-related foods**

Correlation analysis of the global irritable bowel syndrome prevalence, per capita consumption of ginger, and IBS-related foods according to Manning and Rome I–IV criteria (Data reference: Table S1).


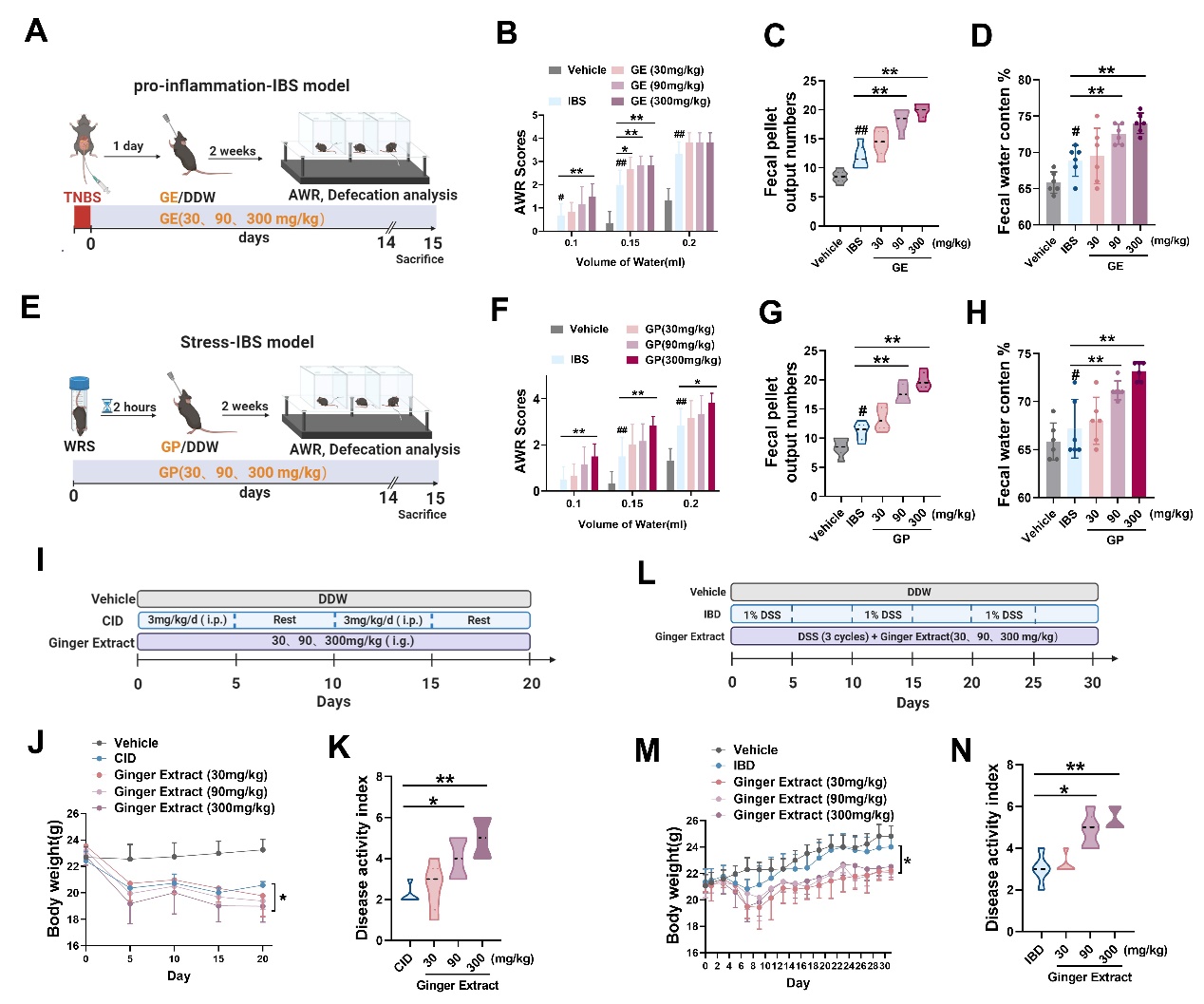


**Fig. S2. Disease-exacerbating effect of dietary ginger in pro-inflammation related IBS model, stress-induced IBS model, chemotherapy-induced irritable bowel model (diarrhea) and DSS-induced chronic colitis model**

(A) Flow chart of the pro-inflammation-related IBS-application model to detect the IBS-aggravating effect of daily ginger consumption (GE, ginger extract). Comparative analysis of the IBS disease index: (B) abdominal withdrawal reflex (AWR) score, (C) number of fecal pellets, and (D) fecal water content of mice after gavage with different doses of GE (according to human daily per capita intake). (E) Flow chart of the stress-induced IBS-application model to detect the IBS-aggravating effect of daily ginger consumption (GP, ginger powder). Comparative analysis of the IBS disease index: (F) abdominal withdrawal reflex (AWR) score, (G) number of fecal pellets, and (H) fecal water content of mice after gavage with different doses of GP (according to human daily per capita intake). (I) Flow chart of the application of chemotherapy (oxaliplatin)-induced irritable bowel model (diarrhea) to detect symptoms of the aggravating effect of ginger. Comparative analysis of (J) body weight and (K) disease activity index after gavage with daily per capita intake of ginger. (L) Flow chart of the application of DSS-induced chronic colitis model to detect IBS symptoms of the aggravating effect of ginger. Comparative analysis of (M) body weight and (N) disease activity index after gavage with daily per capita intake of ginger. Data are expressed as mean ± standard deviation (n = 6). *P < 0.05, **P < 0.01, as indicated; #P < 0.05, ##P < 0.01, compared to vehicle.

DDW, double distilled water; DSS, dextran sodium sulfate; GE, ginger extract; GP, ginger powder; HE, hematoxylin and eosin; IBS, irritable bowel syndrome.


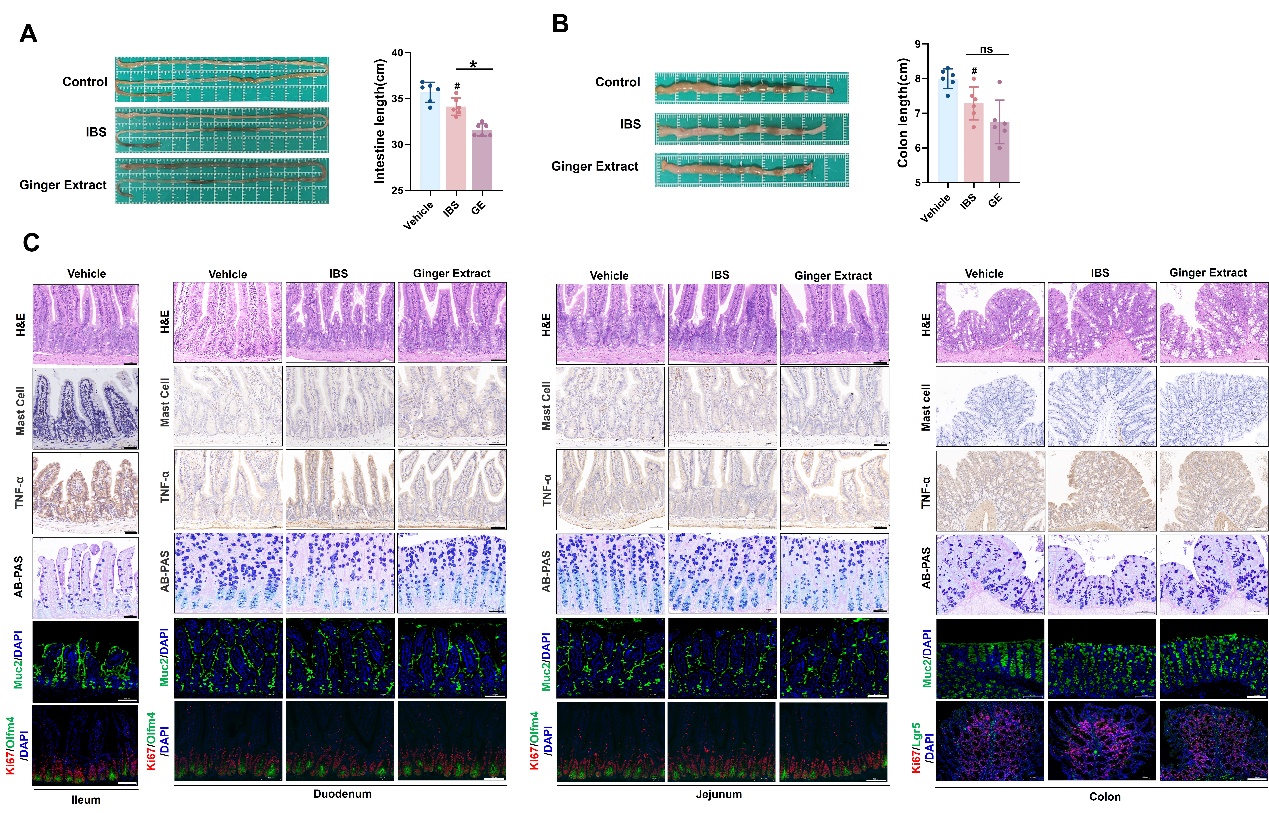


**Fig. S3.** **Effect of dietary ginger extract on the intestinal tract**

(A) Length analysis of mouse intestinal tissues in vehicle, IBS, and ginger extract (GE, 300 mg/kg) groups. (B) Length analysis of mouse colon tissue in vehicle, IBS, and GE groups. (C) HE and AB-PAS staining analysis of mouse intestinal tissues (the duodenum, jejunum, ileum, and colon), immunohistochemical analysis of mast cells, and TNF-α and immunofluorescence analysis of mouse intestinal tissues (the duodenum, jejunum, ileum, and colon) for MUC2, Ki67, and Olfm4 in vehicle, IBS, and GE groups. Scale bar (black) = 50 μm, scale bar (white) = 100 μm. Data are expressed as mean ± standard deviation (n = 6). *P < 0.05, **P < 0.01, as indicated.

HE, hematoxylin and eosin; IBS, irritable bowel syndrome.


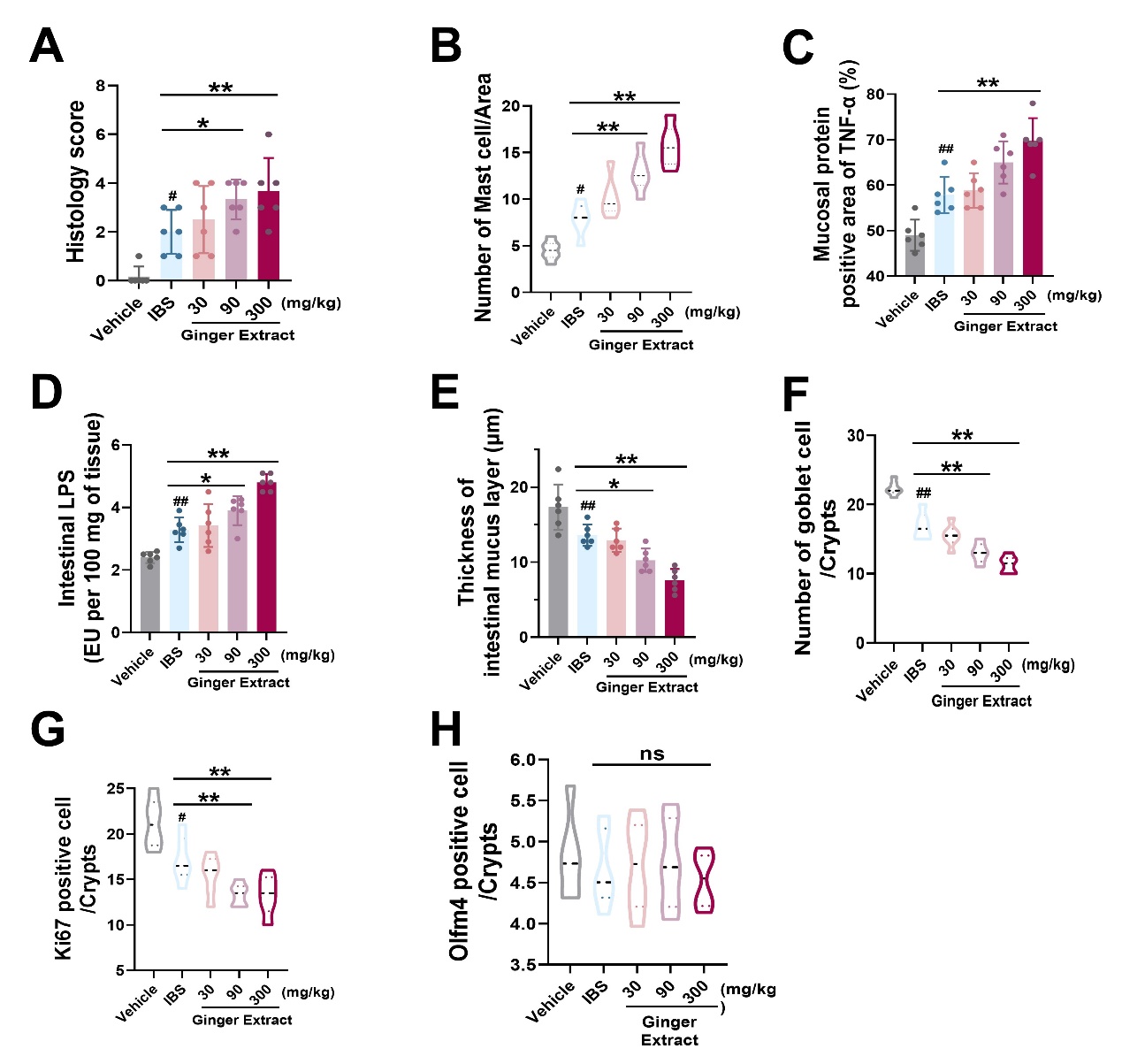


**Fig. S4. Dose-dependent regulation of ileal crypt inflammatory infiltration and stem cell differentiation by dietary ginger**

Comparative analysis of intestinal section (ileum) (A) histology score, (B) mast cell infiltration, (C) TNF-α abundance, (D) intestinal LPS level, (E) thickness of intestinal mucus layer, and (F) mean number of goblet cells, (G) Ki67, (H) Olfm4-positive cells per unit intestinal crypt in vehicle, IBS, and group administered with different dietary dose of ginger. Data are expressed as mean ± standard deviation (n = 6). *P < 0.05, **P < 0.01, compared with IBS.

GE, ginger extract; IBS, irritable bowel syndrome; Olfm4, Olfactomedin 4; WRS, wrap restraint stress.


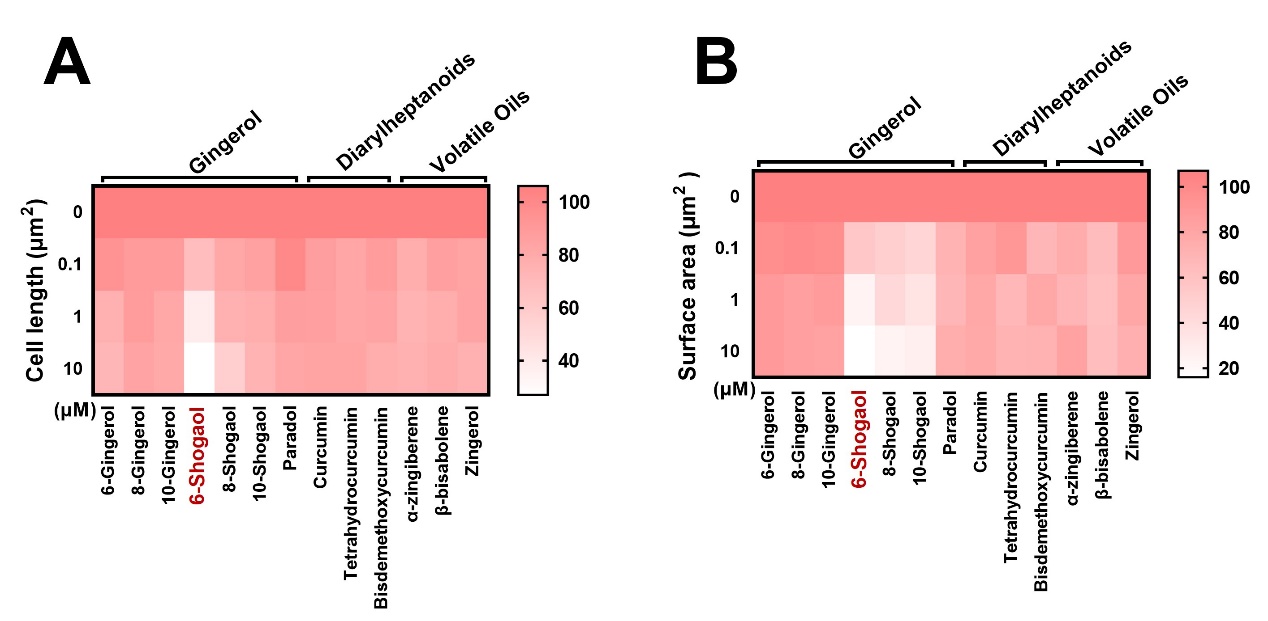


**Fig. S5. Analysis of the main toxic substances in ginger using an** **intestinal organoid differentiation screening model**

Flow diagram of the screening of ginger toxic by-products using an intestinal organoid differentiation model. Screening analysis of the main active components of ginger against the (A) maximum diameter length and (B) average surface area of organoid.


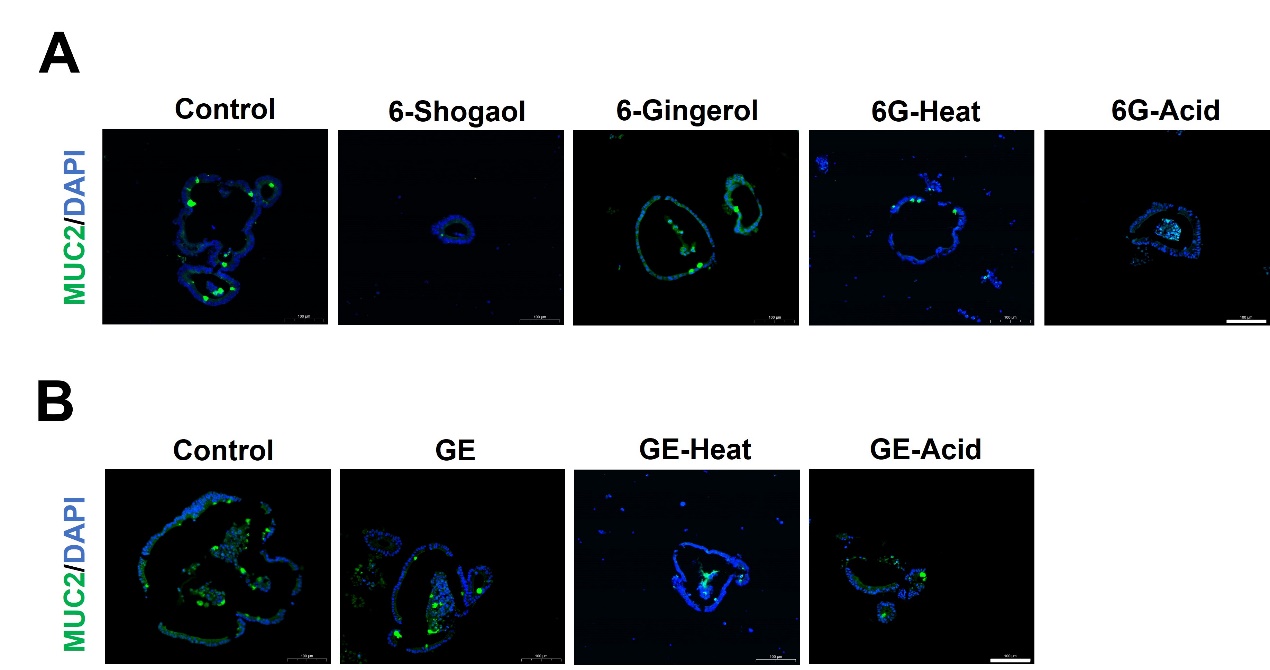


**Fig. S6.** **Analysis of the inhibitory effects of ginger components and ginger extract on the differentiation of goblet cells in intestinal organoids.**

Immunofluorescence analysis of (A) ginger components (6-shogaol and 6-gingerol, each at 1 μM) and (B) ginger extract (3 mg/ml) under heat and acid treatment on goblet cell differentiation in intestinal organoids using MUC2. Scale bar (white) = 100 μm.

GE, ginger extract; 6G, 6-gingerol; MUC2, mucin 2;


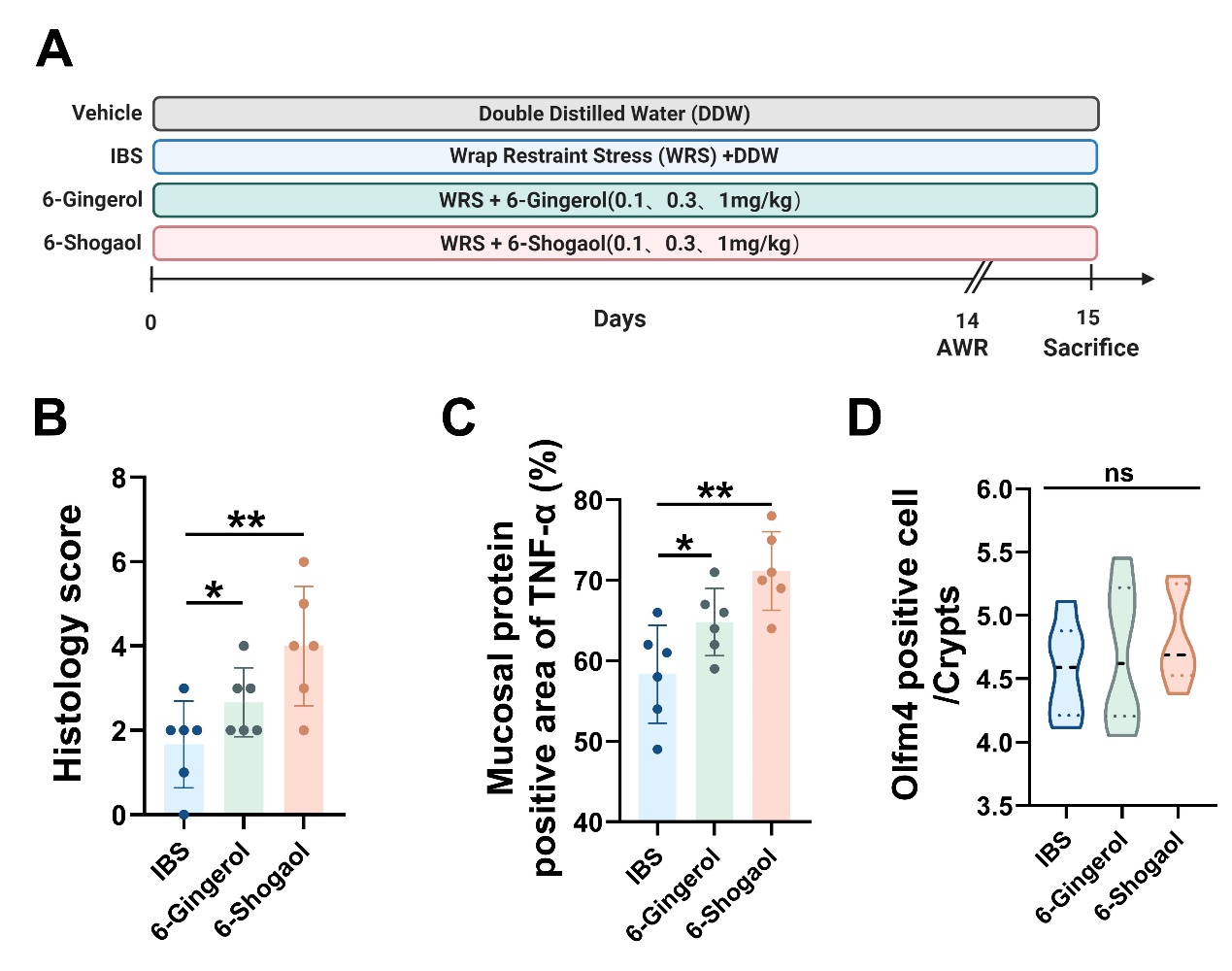
**Fig. S7.** **Infiltration of enteritis was aggravated by 6-shogaol by inhibiting stem cell differentiation**

(A) Flow chart of the application of stress-induced IBS mice model to verify the IBS-aggravating effect of 6-gingerol and 6-shogaol. Comparative analysis of the intestinal section (ileum) (B) histology score, (C) TNF-α abundance, and (I) Olfm4-positive cells per unit intestinal crypt in vehicle, IBS, 6-gingerol (1 mg/kg), and 6-shogaol (1 mg/kg) groups. Data are expressed as mean ± standard deviation (n = 6). *P < 0.05, **P < 0.01, as indicated.

DDW, double distilled water; IBS, irritable bowel syndrome; MUC2, mucin 2; Olfm4, olfactomedin 4.


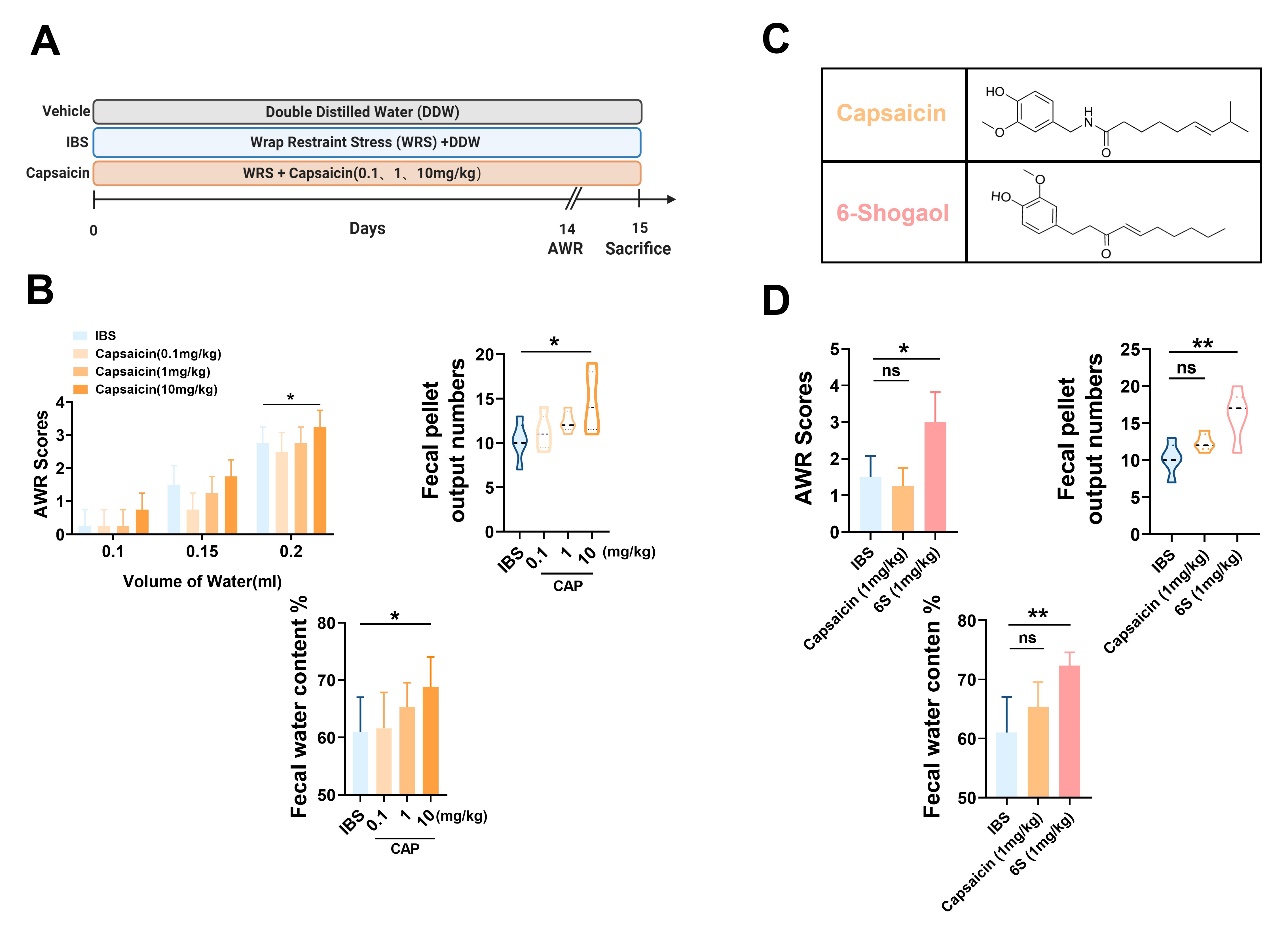
**Fig. S8.** **Comparative analysis of capsaicin and 6-shogaol aggravating the disease index of IBS mice model**

(A) Flow chart of the application of stress-induced IBS mouse model to verify the IBS-aggravating effect of capsaicin. Comparative analysis of the (B) abdominal withdrawal reflex (AWR) score, number of fecal pellets, and fecal water content of mice after gavage with different doses of capsaicin. (C) Chemical structures of capsaicin and 6-shogaol. (D) Comparative analysis of the abdominal withdrawal reflex (AWR) score, number of fecal pellets, and fecal water content of mice after gavage with different doses of capsaicin (1 mg/kg) and 6-shogaol (1 mg/kg). Data are expressed as mean ± standard deviation (n = 6). *P < 0.05, **P < 0.01, as indicated.

DDW, double distilled water; WRS, wrap restraint stress; IBS, irritable bowel syndrome

**
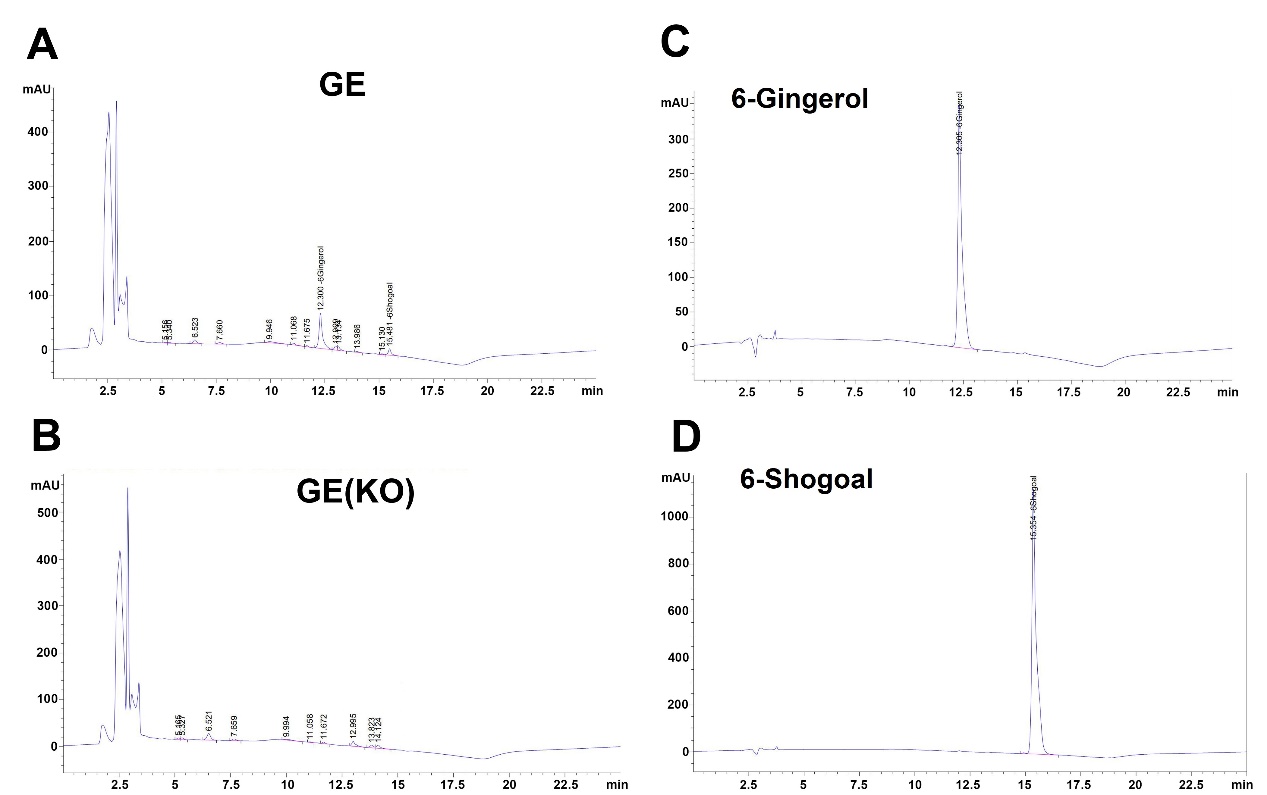
**

**Fig. S9. HPLC analyses of ginger extract component knockout**

(A, B) HPLC analysis of GE and GE (KO). (C, D) HPLC analysis of standard 6-gingerol and 6-shogaol.

GE, ginger extract; GE (KO), ginger extract (6-gingerol and 6-shogaol knockout).


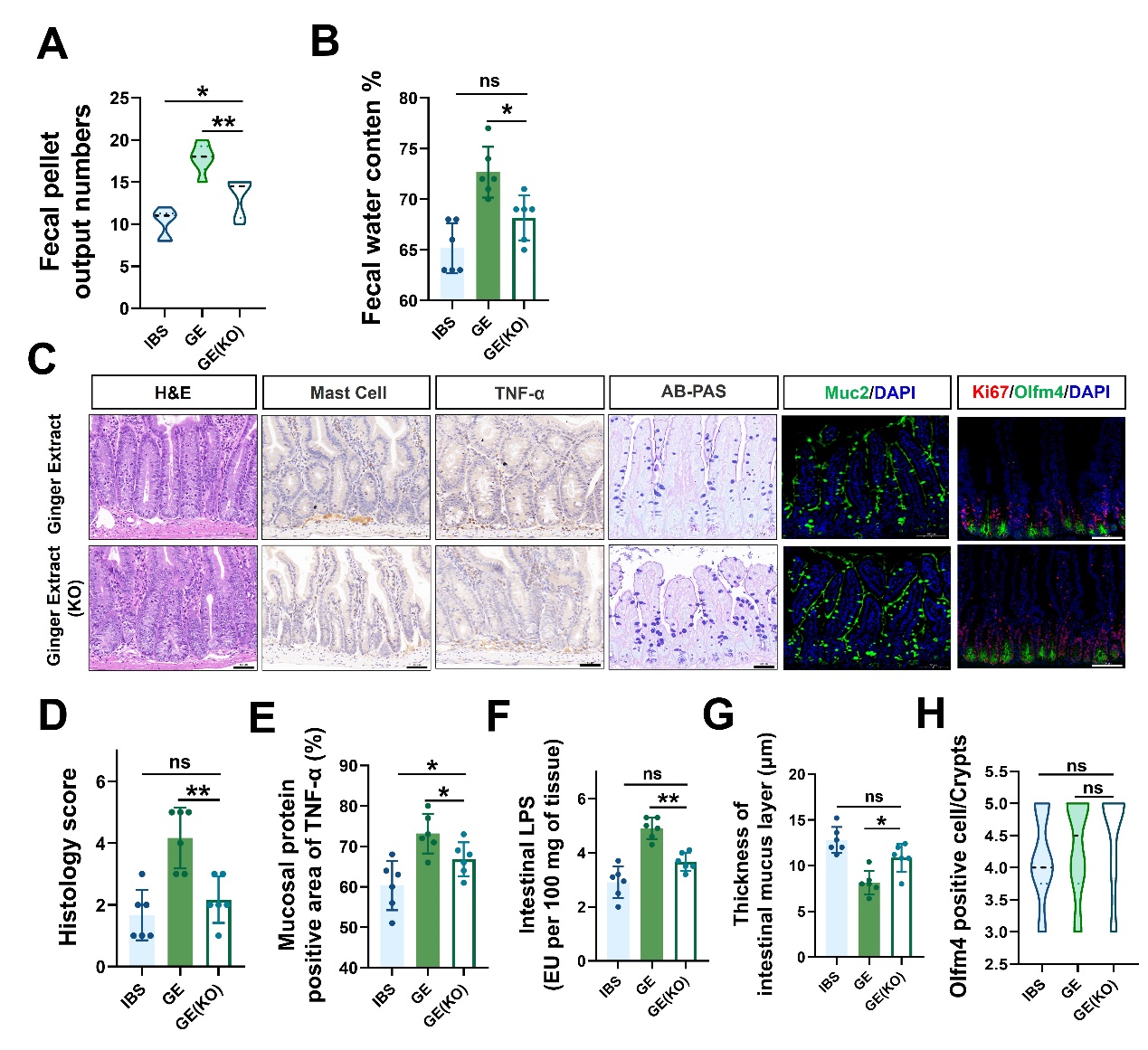


**Fig. S10. Components knockout method analysis of dietary ginger derived-6-shogaol in exacerbated ileal crypt inflammatory infiltration and stem cell differentiation**

Component knock-out method to verify the IBS exacerbation components (6-gingerol and 6-shogaol) in ginger. (A) Comparative analysis of fecal water content and (B) fecal pellet count of GE (ginger raw materials 300 mg/kg) and GE (KO) (ginger raw materials 300 mg/kg) groups. (C) HE and AB-PAS staining analysis of mouse intestinal tissues (ileum), immunohistochemical analysis of mast cell and TNF-α, and immunofluorescence analysis of mouse intestinal tissues for MUC2, Ki67, and Olfm4 in the GE and GE (KO) groups. Scale bar (black) = 50 μm, scale bar (white) = 100 μm. Comparative analysis of intestinal section (ileum) (D) histology score, (E) TNF-α abundance, (F) intestinal LPS level, and (G) thickness of intestinal mucus layer and (H) mean number of Olfm4-positive cells per unit intestinal crypt in GE and GE(KO) groups. Data are expressed as mean ± standard deviation (n = 6). *P < 0.05, **P < 0.01, as indicated.

AB-PAS, alcian blue-periodic acid-Schiff; AWR, abdominal withdrawal reflex; DDW, double distilled water; GE, ginger extract; GE(KO), ginger extract (6-gingerol and 6-shogaol knock-out); HE, hematoxylin and eosin; IBS, irritable bowel syndrome; MUC2, mucin 2; Olfm4, Olfactomedin 4; WRS, wrap restraint stress.


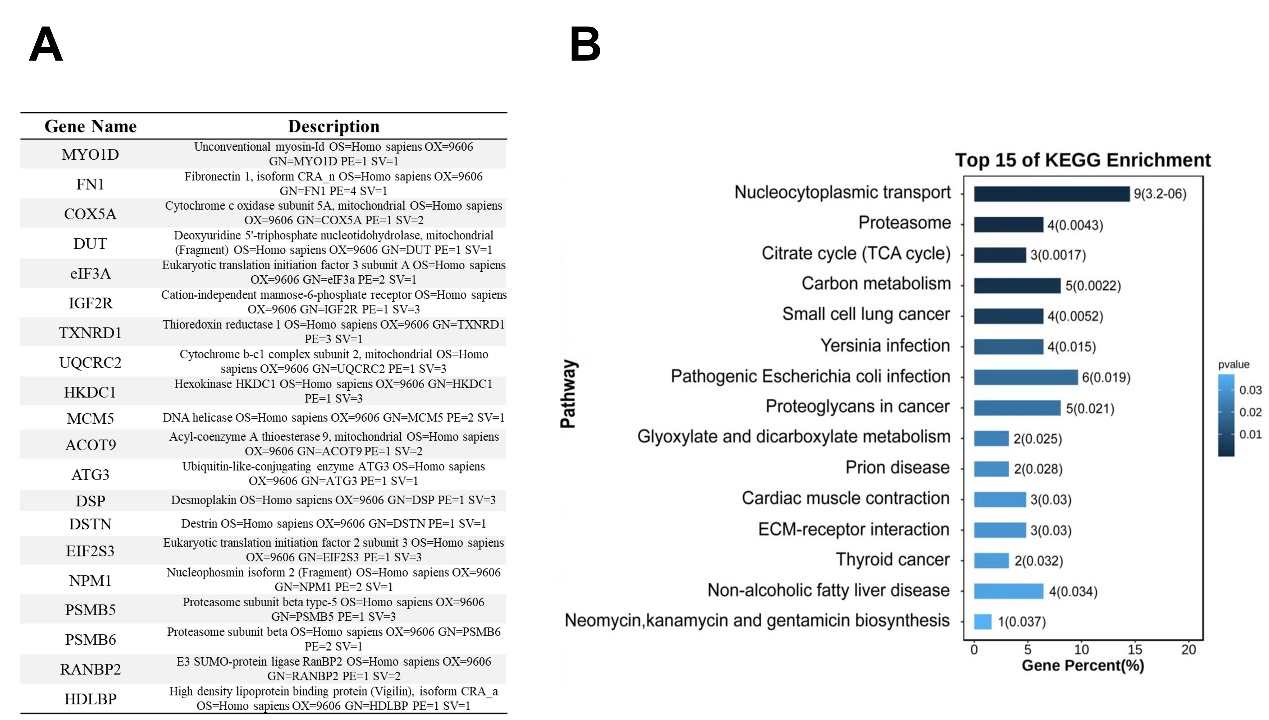


**Fig. S11. Analysis of 6-shogaol binding target proteins**

(A) List of 6-shogaol binding target proteins. (B) KEGG enrichment analysis of the pooled pathways of 6-gingerol binding target proteins.

**Materials and Methods**

**Reagents and antibodies**

Alcian blue-periodic acid-Schiff (AB-PAS), CuSO_4_ (C1297), tris ((1-benzyl-4-triazolyl) methyl) amine (678937), and azo-biotin-azide (900891) were purchased from Sigma-Aldrich (USA). Immunoprecipitation lysis buffer, Dynabeads™ MyOne™ Streptavidin T1, and tris (2-carboxyethyl) phosphine were purchased from Thermo Scientific (USA). Radioimmunoprecipitation assay lysis buffer and alkaline phosphatase assay reagents were purchased from Beyotime (China). The antibodies against the following proteins were used: Anti-Ki67 (ab279657), anti-TNF-alpha (ab183218), anti-mast Cell [AA1] (ab2378), Anti-MUC2 (ab272692) and anti-eIF3A (ab128996) (Abcam, USA); Anti-Olfm4 (#39141T) and anti-Flag (#14793) (Cell Signaling Technology, USA); Anti-eIF3B (sc-137214) (Santa Cruz Biotechnology, USA). Alexa Fluor^®^ 594 streptavidin and Alexa Fluor^®^ 488 were purchased from Invitrogen (Carlsbad, CA, USA). Goat anti-rabbit and anti-mouse IgG antibodies were purchased from LI-COR (Lincoln, NE, USA).

**Lentivirus infection**

To verify the binding target specificity of 6-shogaol and eukaryotic initiation factor (eIF3A), lentivirus (LV) vectors for eIF3A overexpression (LV-flag-eIF3A) and an eIF3A mutant (LV-Flag-eIF3a (C58A)) were constructed. Intestinal crypts isolated from C57BL/6 mice were dissociated into single cells and incubated with the lentiviral particles in Matrigel [4].

**Animal model**

All procedures involving animals were approved by the institutional animal care and use committee of the Jiangsu Province Institute of Traditional Chinese Medicine; the procedural details were drafted in accordance with the ARRIVE guidelines. Experiments were performed in accordance with the guidelines published by the National Institutes of Health. Mice were 10–12 weeks old and weighed 24–26 g at study initiation. For the pro-inflammation IBS model, mice were fasted overnight with access to a 5% glucose solution. After the fasting period, isoflurane-anesthetized mice were administered an intracolonic enema of 0.1 mL of TNBS (6.5 mg in 30% ethanol) via a polyethylene catheter inserted 3 cm past the anus [5]. For ginger exacerbated IBS studies, 30–300 mg/kg ginger extract (equivalent to a daily human dose of 0.2–2 g per person based on ginger daily consumption data, the dosage used in clinical trials, and equivalent dose ratio converted from body surface area [2, 6]) were administered daily by gavage to the mice in the pro-inflammation IBS model for two weeks. For dextran sodium sulfate (DSS) induced chronic colitis model, IBD was induced in mice by administering 1% DSS (w/v) solution in distilled water for 5 days followed by 5 days rest for three cycles. For the chemotherapy-induced diarrhea model, the mice were treated daily with an intraperitoneal injection using volumes of 10 mL/kg for 5 days, followed by 5 days of rest for two-week cycles. A total cumulative dose of 30 mg/kg OHP was used over 10 injections.

After administration of the ginger extract and solvent control, the body weight and intestinal disease activity index of mice were recorded.

**Adeno-associated virus (AAV) infection**

AAV7-mCherry, AAV7-eIF3A, and AAV7-eIF3A(C58A) were prepared by Obio Technology Co., Ltd. (Shanghai, China). Viral titer was 2.31 × 10^13^ vg/mL for AAV7-mCherry, 1.43 × 10^13^ vg/mL for AAV7-eIF3A, and 1.56 × 10^13^ vg/mL for AAV7-eIF3A (C58A). Mice were divided randomly into groups for the eIF3A over-expression experiment, with each group receiving either AAV7-mCherry, AAV7-eIF3A, or AAV7-eIF3A(C58A) via caudal vein injection. The mice were further treated with restraint stress to construct the IBS model [7]. After GE treatment, the fecal water content, fecal pellet output, and visceral sensitivity were measured in each group [8]. The abdominal withdrawal reflex (AWR) was semi-quantitatively scored as previously described [9].

**Human colonic epithelial cell (HCoEpiC) culture**

HCoEpiCs were grown in a colonic epithelial cell medium (cat. #2951) supplemented with the accompanying supplements, as recommended by the manufacturer (ScienCell Research Laboratory, CA, USA). Tissue culture flasks were incubated with poly L-lysine solution (2 μg/cm^2^) at 37 ºC overnight. HCoEpiC were seeded in coated flasks and cultured at 37 ºC and in 5% CO_2_. Confluent cell monolayers (approximately 80%) were used for the assays.

**Histological staining (HE), immunohistochemistry (IHC), and immunofluorescence staining (IF)**

For histological staining, the small intestine tissues from mice in each group were collected and fixed in 4% buffered formalin for 24 h, dehydrated, embedded in paraffin, and cut into approximately 5 μm-thick sections. Subsequently, the tissue sections were stained with hematoxylin and eosin (HE) to evaluate the degree of inflammation in the small intestine and colon and stained with AB-PAS to identify goblet cells in the epithelium, small intestine, and colon [10-13].

For IHC, the degree of inflammation in the small intestine was assessed by detecting the expression of mast cells and TNF-α [14, 15]. The expression levels of mast cells and TNF-α in paraffin-embedded sections (4 μM) of intestinal tissues were evaluated using IHC with specific antibodies. After dewaxing, the sections underwent antigen repair, background blocking, and incubation with primary antibodies at 4 °C overnight. Biotin-labeled secondary antibodies and streptavidin-horseradish peroxidase were incubated at 20 °C for 30 min. Immune responses were detected using 3-3-diaminobenzidine and counterstained with hematoxylin. The sections were observed under an optical microscope.

For IF, sections were permeabilized with 0.2% Triton X-100 in phosphate-buffered saline (PBS) for 20 min and blocked in PBS containing 1% bovine serum albumin (BSA) for 1 h to reduce non-specific binding. The sections were then incubated with primary antibodies overnight at 4 °C, rinsed with PBS, and incubated with secondary antibodies for 2 h at 20 °C in the dark.

After three washes with PBS, the sections were incubated with 4′, 6-diamidino-2-phenylindole dihydrochloride (DAPI) for 5 min and washed thrice with PBS. The slides were mounted and examined under a Nikon A1 confocal laser microscope (Nikon, Tokyo, Japan) [16]. The average mucus thickness was detected and calculated from the immunofluorescence thickness of muc2 protein [17]. The average number of Ki67 proliferating cells (transit-amplifying zone-Ki67+) and Olfactomedin (Olfm) 4-positive stem cells were calculated from the number of Ki67+ and Olfm-4-positive cells in each crypt, respectively [18].

**IF of the organoids**

After medium removal, the organoids were fixed in 4% paraformaldehyde. Permeabilization was performed with 0.2% Triton X-100, followed by blocking with 5% BSA. The organoids were incubated with anti-Ki67 (ab279657) or anti-MUC2 (ab272692) at 4 °C overnight. The organoids were incubated with secondary antibodies, either Alexa Fluor 488-conjugated anti-mouse IgG (Life Technologies, MD, USA) or Alexa Fluor 555-conjugated anti-rabbit IgG (Life Technologies), at 20 °C for 2 h. Nuclei were stained with DAPI (Sigma-Aldrich) for 1 h, and the organoids were imaged using a confocal microscope (FV10i, Olympus, Japan). The mean fluorescence intensity was analyzed using the ImageJ software, and the intensity of each marker was normalized to that of DAPI.

**Western blotting**

Total protein was isolated using a lysis buffer containing protease inhibitors. Absorbance was measured using a NanoDrop 1000 spectrophotometer (Thermo Fisher Scientific). Protein samples were separated on 10% sodium dodecyl sulfate-polyacrylamide gels (SDS-PAGE) and transferred onto hydrophilic polyvinylidene fluoride membranes (Millipore, USA). After blocking with 1% BSA in tris buffered saline + tween 20 (TBST) for 2 h, the membrane was incubated with primary antibodies overnight at 4 °C, followed by a secondary antibody for 1 h at 20 °C. Membranes were washed three more times with TBST, scanned with an Odyssey Infrared Fluorescence Scanner (LI-COR), and analyzed using the Odyssey software version 3 [19].

**Lipopolysaccharide (LPS) assay**

All materials used for sample preparation and testing were pyrogen-free. LPS concentrations in plasma and tissue homogenate were measured by an endotoxin assay based on a Limulus amebocyte extract with a chromogenic limulus amebocyte lysate (LAL) assay (Pierce LAL Chromogenic Endotoxin Quantitation Kit). Samples were diluted in pyrogen-free water and heated to 70 °C for 10 min to inactivate inhibitor agents that could interfere with the assay. All samples were tested in triplicate, and results were accepted when the intra-assay coefficient of variation was 15%. The endotoxin content was expressed as endotoxin units per milliliter (EU/mL) or per 100 mg of tissue [20].

**Knock out of the target components**

Under the optimal conditions of high-performance liquid chromatography (HPLC), 5 mL of ginger extract (equivalent to 1 g/mL of the raw material) was injected every time under the optimized chromatography conditions. The eluent solution of the target components (6-gingerol and 6-shogaol) and the negative solution without the target components (6-gingerol and 6-shogaol) were collected according to the peak retention time from the spectrum-effect relationship analysis, respectively. Each component was prepared and eluted in a 15-fold series. The filtrated solution contained the target component (denoted as GE) and corresponding negative sample (denoted as GE (KO)) as previously described [21, 22].

**Non-denaturing gel electrophoresis**

Protein samples were diluted with Native PAGE sample buffer and electrophoresed on a 4–20% Tris-Glycine gel (Life Technologies) at 100 V for 24 h at 4 °C. The proteins were transferred onto nitrocellulose membranes and probed with antibodies. Membranes were then extensively washed and incubated with primary antibody overnight at 4 °C, followed by secondary antibody for 1 h at 20 °C. Membranes were washed three more times with TBST, scanned with an Odyssey Infrared Fluorescence Scanner (LI-COR), and analyzed using Odyssey software version 3.

**Synthesis of 6-shogaol probe (6S-1)**

Charge anticoagulant citrate phosphate dextrose solution (cpd.) 1 (2.1 g, 10 mmol), imidazole (1.7 g, 25 mmol), and dichloromethane (DCM) (20 mL) in a 50 mL 3-neck flask with nitrogen protection were cooled down to 0–5 °C. Charged tert-Butyldimethylsilyl chloride (3.0 g, 20 mmol) was then added to the flasks maintained at 0–5 °C. The reaction mixture was heated to 20–30 °C naturally. The mixture was stirred at 20–30 °C (IT) for 6 h. The reaction was doused with methanol (4 mL). Charged water (40 mL) was then added to the mixture. The two phases were separated. The aqueous phase was extracted using DCM (20 mL × 2). All the DCM phases were combined and washed with brine (26.5 w%, 20 mL). The solution was concentrated to dryness. The crude was purified using cpd. 2 by column to obtain 2.9 g of product two with 90% yield.

A solution of dimethyl methyl phosphonate (1.08 mL, 10.0 mmol) in anhydrous tetrahydrofuran (THF) (12.5 mL) was charged in a 50 mL three-neck flask with nitrogen protection. We added n-Butyllithium (6.41 mL, 1.56 M in hexane) using a syringe at -65 °C, and the reaction was stirred for 15 min. A solution of cpd. 2 (3.24 g, 10.0 mmol) in dry THF (2.5 mL) was added using a syringe while maintaining an internal temperature below -65 °C. The reaction mixture was stirred for 15 min and treated with p-toluenesulfonic acid monohydrate (1.90 g, 10.0 mmol) in a single portion. The cooling bath was removed to effect acid dissolution, which was complete at -5 °C. At 20 °C, the sample was concentrated in vacuo until dryness. Dissolution in methanol and direct loading onto a silica column produced 2.8 g of cpd. 3 with 67% yield.

A 10 mL three-neck flask was charged with 1, 4-dioxane (2.0 mL), water (54 μL, 3.0 mmol), cpd. 3 (1.04 g, 2.5 mmol), cpd. 4 (308 mg, 2.0 mmol), and cesium carbonate (978 mg, 3.0 mmol). The resulting heterogeneous reaction mixture was then stirred at 20 °C for 3 h. The reaction mixture was filtered through a 15 mL medium porosity fritted glass disc filter covered by a 2.0 cm thick layer of Celite® 545, and the filter cake was washed with DCM (10 mL × 3). The solvent was evaporated under reduced pressure to yield a light-oily residue. Ca. 633 mg of cpd. 5 was obtained as a solid following flash column chromatography on silica gel with a 69% yield.

We charged cpd. 5 (460 mg, 1.0 mmol) and THF (1.5 mL) in a 10 mL flask under nitrogen. Triethylamine trihydrofluoride (322 mg, 2.0 mmol) was slowly added to the reactor. The mixture was stirred at 20–30 °C (IT) for 24 h. The reaction mixture was quenched with aq. NH_4_Cl (37 w%, 25 mL) and stirred for 5 min. The two phases were then separated. The aqueous phases were cooled to 0–5 °C and neutralized with aq. NaHCO_3_ (7.2 %, 7.5 mL) to pH ~6–7.9. The aqueous phase was extracted using DCM (2.5 mL × 3). All organic phases were combined. The combined organic phases were concentrated until dryness. The crude TM was purified by column to obtain 220 mg of product TM with a 64 % yield. We purified and analyzed 6-shogoal probes using high-performance liquid chromatography and mass spectrometry, respectively.

**Immunoprecipitation**

The culture medium was aspirated, washed once with PBS, and the cells were lysed by adding cell lysate. We added IgG and fully resuspended Protein A+G Agarose, incubated the medium at 4 °C for 1 h, centrifuged for 5 min (2,500 rpm), and took the supernatant. We added primary antibody and incubated at 4 °C overnight. Further, we added fully resuspended Protein A+G Agarose and incubated it at 4 °C for 2 h. After centrifugation for 5 min, the supernatant was removed by suction, and the precipitate was washed with PBS five times.

**Mass spectrometry analysis**

HCoEpiC cells or small intestine organoid lysates were incubated with 10 μM 6-shogoal probe or dimethylsulfoxide (negative control) for 4 h and lysed by adding the cell lysate. The lysate was labeled with biotin azide and affinity enriched using avidin beads. Beads were washed thoroughly and trypsinized, and labeled samples were pooled, identified using liquid chromatography-tandem mass spectroscopy, and screened for proteins of interest. For the 6-shogoal-eIF3A complex assay, small intestinal organoids were infected with LV-flag-eIF3A. The cells were incubated with 6-shogoal (10 μM) for 24 h. eIF3A proteins were purified from cell lysates using the anti-FLAG Ab-conjugated agarose. Eluted protein samples were reduced with dithiothreitol, diluted with low concentrations of RapiGest SF without urea, and digested with trypsin. Finally, the tryptic digests were desalted and renatured prior to matrix-assisted laser desorption/ionization-time of flight analysis (MALDI-7090, Shimadzu Kratos) as previously described [23, 24].

**Kyoto Encyclopedia of Genes and Genomes (KEGG) enrichment analyses**

To identify the pathways of the toxic components of ginger, we conducted a KEGG enrichment analysis. The KEGG database contains information on various biological pathways. KEGG enrichment analysis revealed the pathways in which differentially expressed genes were involved, which can indicate the molecular mechanism of a disease [25]. In this study, statistical significance was set at p < 0.05. A total of 66 pathways were screened, and the top 20 pathways were selected.

**References**

[1] Lovell RM, Ford AC. Global prevalence of and risk factors for irritable bowel syndrome: A meta-analysis. *Clin Gastroenterol Hepatol*. 2012;10:712-721 e714.

[2] INDEXBOX. Ginger-world. [*https://appindexboxio/table/091010h091012/0/*](https://appindexboxio/table/091010h091012/0/)

（accessed April 2023).

[3] Sperber AD, Bangdiwala SI, Drossman DA, Ghoshal UC, Simren M, Tack J, Whitehead WE, Dumitrascu DL, Fang X, Fukudo S, et al. Worldwide prevalence and burden of functional gastrointestinal disorders, results of rome foundation global study. *Gastroenterology*. 2021;160:99-114 e113.

[4] Onuma K, Ochiai M, Orihashi K, Takahashi M, Imai T, Nakagama H, Hippo Y. Genetic reconstitution of tumorigenesis in primary intestinal cells. *Proc Natl Acad Sci U S A*. 2013;110:11127-11132.

[5] Jiang Y, Castro J, Blomster LV, Agwa AJ, Maddern J, Schober G, Herzig V, Chow CY, Cardoso FC, Demétrio De Souza França P, et al. Pharmacological inhibition of the voltage-gated sodium channel na(v)1.7 alleviates chronic visceral pain in a rodent model of irritable bowel syndrome. *ACS pharmacology & translational science*. 2021;4:1362-1378.

[6] Anh NH, Kim SJ, Long NP, Min JE, Yoon YC, Lee EG, Kim M, Kim TJ, Yang YY, Son EY, et al. Ginger on human health: A comprehensive systematic review of 109 randomized controlled trials. *Nutrients*. 2020;12.

[7] Xu D, Gao J, Gillilland M, 3rd, Wu X, Song I, Kao JY, Owyang C. Rifaximin alters intestinal bacteria and prevents stress-induced gut inflammation and visceral hyperalgesia in rats. *Gastroenterology*. 2014;146:484-496 e484.

[8] Ke W, Wang Y, Huang S, Liu S, Zhu H, Xie X, Yang H, Lu Q, Gan J, He G, et al. Paeoniflorin alleviates inflammatory response in ibs-d mouse model via downregulation of the nlrp3 inflammasome pathway with involvement of mir-29a. *Heliyon*. 2022;8:e12312.

[9] Zhang Y, Zhang H, Zhang W, Zhang Y, Wang W, Nie L. Lncrna xist modulates 5-hydroxytrytophan-induced visceral hypersensitivity by epigenetic silencing of the sert gene in mice with diarrhea-predominant ibs. *Cell Signal*. 2020;73:109674.

[10] Yang Y, Li L, Hang Q, Fang Y, Dong X, Cao P, Yin Z, Luo L. Gamma-glutamylcysteine exhibits anti-inflammatory effects by increasing cellular glutathione level. *Redox Biol*. 2019;20:157-166.

[11] Sun X, Pisano M, Xu L, Sun F, Xu J, Zheng W, Liu X, Zhang Y, Sun R, Cui X. Baicalin regulates autophagy to interfere with small intestinal acute graft-versus-host disease. *Sci Rep*. 2022;12:6551.

[12] Song S, Bai M, Ling Z, Lin Y, Wang S, Chen Y. Intermittent administration of a fasting-mimicking diet reduces intestinal inflammation and promotes repair to ameliorate inflammatory bowel disease in mice. *J Nutr Biochem*. 2021;96:108785.

[13] Cui H, Cai Y, Wang L, Jia B, Li J, Zhao S, Chu X, Lin J, Zhang X, Bian Y, et al. Berberine regulates treg/th17 balance to treat ulcerative colitis through modulating the gut microbiota in the colon. *Front Pharmacol*. 2018;9:571.

[14] Li X, Liu Q, Yu J, Zhang R, Sun T, Jiang W, Hu N, Yang P, Luo L, Ren J, et al. Costunolide ameliorates intestinal dysfunction and depressive behaviour in mice with stress-induced irritable bowel syndrome via colonic mast cell activation and central 5-hydroxytryptamine metabolism. *Food Funct*. 2021;12:4142-4151.

[15] Bradford EM, Ryu SH, Singh AP, Lee G, Goretsky T, Sinh P, Williams DB, Cloud AL, Gounaris E, Patel V, et al. Epithelial tnf receptor signaling promotes mucosal repair in inflammatory bowel disease. *J Immunol*. 2017;199:1886-1897.

[16] Yang Y, Yin F, Hang Q, Dong X, Chen J, Li L, Cao P, Yin Z, Luo L. Regulation of endothelial permeability by glutathione s-transferase pi against actin polymerization. *Cell Physiol Biochem*. 2018;45:406-418.

[17] Ermund A, Schütte A, Johansson ME, Gustafsson JK, Hansson GC. Studies of mucus in mouse stomach, small intestine, and colon. I. Gastrointestinal mucus layers have different properties depending on location as well as over the peyer's patches. *Am J Physiol Gastrointest Liver Physiol*. 2013;305:G341-347.

[18] Lee C, Choi C, Kang HS, Shin SW, Kim SY, Park HC, Hong SN. Nod2 supports crypt survival and epithelial regeneration after radiation-induced injury. *Int J Mol Sci*. 2019;20.

[19] Yang Y, Cai X, Yang J, Sun X, Hu C, Yan Z, Xu X, Lu W, Wang X, Cao P. Chemoprevention of dietary digitoflavone on colitis-associated colon tumorigenesis through inducing nrf2 signaling pathway and inhibition of inflammation. *Mol Cancer*. 2014;13:48.

[20] Shen S, Lim G, You Z, Ding W, Huang P, Ran C, Doheny J, Caravan P, Tate S, Hu K, et al. Gut microbiota is critical for the induction of chemotherapy-induced pain. *Nature neuroscience*. 2017;20:1213-1216.

[21] You H, Ireland B, Moeszinger M, Zhang H, Snow L, Krepich S, Takagawa V. Determination of bioactive nonvolatile ginger constituents in dietary supplements by a rapid and economic hplc method: Analytical method development and single-laboratory validation. *Talanta*. 2019;194:795-802.

[22] Li W, Zhang Y, Shi S, Yang G, Liu Z, Wang J, Kang W. Spectrum-effect relationship of antioxidant and tyrosinase activity with malus pumila flowers by uplc-ms/ms and component knock-out method. *Food Chem Toxicol*. 2019;133:110754.

[23] Yan SH, Zhao NW, Geng ZR, Shen JY, Liu FM, Yan D, Zhou J, Nie C, Huang CC, Fang ZY. Modulations of keap1-nrf2 signaling axis by tiia ameliorated the oxidative stress-induced myocardial apoptosis. *Free Radic Biol Med*. 2018;115:191-201.

[24] Dong X, Yang Y, Zhou Y, Bi X, Zhao N, Zhang Z, Li L, Hang Q, Zhang R, Chen D, et al. Glutathione s-transferases p1 protects breast cancer cell from adriamycin-induced cell death through promoting autophagy. *Cell death and differentiation*. 2019;26:2086-2099.

[25] Kanehisa M, Goto S. Kegg: Kyoto encyclopedia of genes and genomes. *Nucleic Acids Res*. 2000; 28:27-30.
